# Epigenetic Aging and Cognitive Performance in General and Psychiatric Populations: A Systematic Review and Narrative Synthesis

**DOI:** 10.1016/j.bpsgos.2026.100726

**Published:** 2026-03-30

**Authors:** Natan Yusupov, Julia Fietz, Vera N. Karlbauer, Isabel Maurus, Peter Falkai, Elisabeth B. Binder

**Affiliations:** a Department Genes and Environment, Max Planck Institute of Psychiatry, Munich, Germany; b Department of Psychiatry and Psychotherapy, University Hospital, Ludwig Maximilian University of Munich, Munich, Germany; c Max Planck Institute of Psychiatry, Munich, Germany; d German Centre for Mental Health, Partner site Munich/Augsburg, Germany; e Graduate School of Systemic Neurosciences, Ludwig Maximilian University of Munich, Munich, Germany

**Keywords:** Cognition, Cognitive decline, Epigenetic age, Epigenetic clocks, Psychiatry, Systematic review

## Abstract

Epigenetic aging and age-related cognitive decline both show substantial variability across individuals. While a potential link between the two is increasingly being studied in both general and psychiatric populations, a systematic review has not yet been performed. This review summarizes the state of the current literature as well as critical evaluation and suggestions for future research. A literature search (October 31, 2024) identified original peer-reviewed studies examining epigenetic aging and cognitive function in general and/or psychiatric populations following a preregistered protocol (PROSPERO Registration No. CRD42024608024). Other article types or non-English language works were excluded. A total of 57 observational studies (*n*_General_ = 51, *n*_Psychiatric_ = 6) were included, with a total number of 37,516 participants in 47 cross-sectional studies and 15,551 participants in 27 longitudinal assessments. Sixteen tools of epigenetic age estimation were investigated, mainly in peripheral blood (*n* = 53). Quality assessment with the Agency for Healthcare Research and Quality checklist showed low risk of bias in 93% of studies. Great heterogeneity existed in cohort characteristics, cognitive tests, epigenetic age assessments, use of covariates, and statistical modeling. Overall, significant associations of cognitive measures with epigenetic aging measures were found in 43 studies, 5 of which included psychiatric patients. Evidence supported a link between epigenetic age acceleration and worse cognitive performance in 21/38 (55%) studies. In conclusion, the current literature shows encouraging results, but studies with psychiatric populations remain scarce. To establish epigenetic aging tools as biomarkers of premature cognitive aging, more research is needed.

## Plain Language Summary

Epigenetic aging and age-related cognitive performance can substantially vary between individuals. Their relationship is increasingly being investigated in both general and psychiatric populations, but a systematic review within these populations in the necessary detail is not available. We systematically reviewed the literature to summarize and assess existing evidence, identify current gaps and future challenges. The evidence supports a link between epigenetic age acceleration and worse cognitive performance, although interpretation is limited due to great methodological heterogeneity. Our work presents a critical evaluation and provides suggestions for future advancements in the field.

Aging is a complex multifactorial process that is not yet fully understood (1). However, it is clear that individuals show different aging trajectories, with substantial variability in the aging process that possibly relates to different aging rates across specific tissues or cells (2,3). Aging itself is considered to promote cognitive deterioration (4) and presents one of the strongest risk factors for neurodegenerative disease (5). Cognitive aging is a process of gradual impairment in cognitive functions over the course of life (6). Although an emerging issue in public health, the underlying neurobiology remains unclear (7). Evidence reported to date has revealed that age-related cognitive decline can be identified from young adulthood onward in multiple cognitive domains including memory, reasoning, executive function, and most strongly processing speed (6,8,9).

Among others, brain diseases can impact cognitive function. Beyond neurodegenerative brain diseases, cognitive dysfunction is a common and important feature of psychiatric disorders with great functional impact on quality of life and occupation (10,11). Across psychiatric disorders, deficits are found in multiple cognitive domains such as executive function, episodic memory, attention, and processing speed (12, 13, 14, 15), with cognitive dysfunction possibly representing a transdiagnostic phenomenon shared by various psychiatric disorders [see (16) for a review].

Epigenetic alterations, and specifically DNA methylation, are hallmarks of aging (1). It could be used to quantify an individual’s degree of aging, referred to as epigenetic age (17,18). As a measure of advanced biological aging, epigenetic age acceleration, i.e., higher biological over chronological age, is an increasingly investigated phenomenon in health and disease (17,18) including psychiatry (19). Increased epigenetic aging has been reported for various psychiatric disorders [e.g., (20, 21, 22)] and linked to premature cognitive aging, detectable even in midlife (23, 24, 25). Therefore, epigenetic age was suggested as a peripheral biomarker for brain health and disease (26). Such a biomarker could assist with early detection of a potential subset of psychiatric patients at increased risk for cognitive dysfunction and decline. This group may benefit from targeted treatment and prevention strategies.

Therefore, we sought to systematically review the literature in general and in psychiatric populations to elucidate the existing evidence on the relationship between cognitive function and epigenetic age. Furthermore, we aim to highlight the gaps that need to be addressed to advance the potential application of epigenetic aging as a biomarker of cognitive function.

## Methods

The review is focused on studies examining the relationship between epigenetic age and cognitive function that included both psychiatric and general nonclinical populations. The report followed the Preferred Reporting Items for Systematic Reviewers and Meta-analysis (PRISMA) 2020 guidelines (27) and was preregistered in the international prospective register of systematic reviews (PROSPERO Registration No. CRD42024608024).

### Search Strategy and Data Sources

A search was conducted on October 31, 2024, using the following electronic databases: PubMed, PsychINFO, and Web of Science Core Collection. The search protocol included the following terms: [(“cognition” OR “cognitive function” OR “cognitive functioning” OR “cognitive ability” OR “cognitive dysfunction” OR “cognitive deficits” OR “cognitive performance” OR “cognitive decline” OR “cognitive impairment” OR “cognitive aging” OR “executive function” OR “executive functioning” OR “attention” OR “inhibition” OR “flexibility” OR “processing speed” OR “memory” OR “concentration” OR “language” OR “learning”) AND (“epigenetic clock” OR “DNA methylation clock” OR “DNA methylation age” OR “epigenetic age” OR “epigenetic aging” OR “epigenetic ageing”)]. All articles were pooled in EndNote X9 (later updated to EndNote 2025) and deduplicated (28).

### Inclusion and Exclusion Criteria

The following inclusion criteria were used: 1) studies of cohorts from the general population and/or patients with psychiatric disorders and 2) studies examining the relationship between one or more epigenetic age estimator and any measure of cognitive function. The following exclusion criteria were used: 1) reviews, meta-analyses, conference abstracts, dissertations, correspondence articles, and case studies; 2) studies not yet published in a peer-reviewed journal; and 3) non-English articles.

### Article Selection

Studies were screened by 2 independent reviewers (NY and JF), and possible conflicts were resolved by experts in the field (EBB, PF). After initial exclusion of studies by screening for title and abstract, articles were fully reviewed (NY and JF/VNK) and assessed for eligibility. Additional articles identified during the reading process that did not come up in the initial search but fulfilled the inclusion criteria were also included (snowballing). Whenever a study population focused on participants with a specific disease (e.g., human immunodeficiency virus), the study was included only if separate information for control participants was available. In that case, a comment was added to Table 1, and only data for control participants were considered as coming from a general population.

**Table 1:** Qualitative Summary of Included Articles

Study (First Author, Year)	Epigenetic Age Estimator	Tissue Type, Measurement Platform	Analysis Design^a^	Cohort, Country, Ethnicity^b^	Sample Size^a^ (Female Sex/Gender, %)^c^	Mean Age in Years (SD, Range)^a^	Cognitive Domains (Tests)	Relevant Outcome; Statistical Model	Relevant Positive Findings^d^	Control Variables	oRoB	Comments
Ware *et al.*, 2025 (34)	Horvath 1 Horvath 2 Hannum PhenoAge GrimAge DunedinPoAm (91)	Blood, EPIC	CS	Subsample from HRS, United States, mixed ethnicities (majority non-Hispanic White)	3585 (NA for specific analysis)	NA for specific analysis	Verbal episodic memory Mental status Reasoning Crystallized intelligence Dementia Fluid intelligence All measures from modified TICS	Overall cognitive performance; weighted multivariable logistic regression models	Higher AgeAccelGrim associated with higher odds of cognitive impairment (nondementia)	Age Sex/gender Ethnicity Cell type composition Educational attainment	Low	Information regarding specific cognitive tests not mentioned by authors Main results for dichotomized variables, some differences for continuous AgeAccel Low education and high AgeAccelGrim/DunedinPoAm presented additive interaction effects on odds of dementia Association of education with dementia mediated by AgeAccelGrim/DunedinPoAm in a 4-way mediation interaction decomposition analysis
Engvig *et al.*, 2025 (82)	Horvath 1 Horvath 2 PhenoAge GrimAge GrimAge2 PCBrainAge (116) DunedinPACE	Blood, EPIC	CS	Subsample of 3 cohorts from the ADNI ADNI1, ADNI2, ADNIGO, United States and Canada, ethnicity not specified	257 (NA for specific analysis)	NA for specific analysis	Verbal episodic memory (Immediate Recall from RAVLT) Executive function (TMT-B)	Verbal episodic memory and executive function; bivariate linear regression models	Higher AgeAccelGrim2 and DunedinPACE nominally associated with worse verbal episodic memory	Age Sex/gender	Low	DunedinPACE and GrimAge2 (combined 1 PC) not beneficial for clinical prediction of dementia-related outcomes
Zavala *et al.*, 2024 (33)	Horvath 1 (87) Horvath 2 (117) Hannum (88) PhenoAge (89) GrimAge (90)	Blood, EPIC	LONGL (14 days)	Subsample from ESCAPE study, United States, mixed ethnicities (majority non-Hispanic Black, 58%)	142 (65%)	47 (11,25–65)	Processing speed (Symbol Search, smartphone-based) Working memory (Dot Memory, n-Back, smartphone-based)	Average level and intraindividual variability in daily life in each of following cognitive domains: processing speed and working memory; multilevel models	Higher DeltaAgeHorvath1/2 associated with lower mean processing speed over time Higher DeltaAgeGrim associated with lower mean working memory over time Higher DeltaAgeHorvath1 associated with higher intraindividual variability in processing speed over time Higher DeltaAgePheno associated with lower intraindividual variability in processing speed over time Higher DeltaAge of all measurements associated with higher intraindividual variability in working memory over time	Sex/gender Education Smoking status (except for GrimAge) Genetic ancestry (PCs) Practice with tasks (linear and quadratic effects)	Low	DNAmAge estimated using PC clocks (114) Age acceleration defined as DNAmAge − chronological age Cognitive performance in daily life evaluated 5 quasirandom times per day
Wolf *et al.*, 2024 (23)	GrimAge	Blood, EPIC	LONGL (mean: ∼6 y)	Trauma-exposed military veterans (majority with lifetime PTSD), United States, mixed ethnicities (majority White)	159 (15%)^f^	T1: 53 (12) f T2: 59 (12,28–78) f	Working memory (DSF/DSB from WAIS-IV) Verbal episodic memory (Short, Long Delay Free Recall and Recognition from CVLT-II) Executive function/attention (Color-Word Interference Test, Letter Fluency, Category Fluency, Category Switching from Delis-Kaplan Executive Function System, TMT-A/B)	Latent variables (from CFA) at T2 representing working memory, executive function/attention, and verbal episodic memory; cross-lagged panel mediation models	Higher AgeAccelGrim at baseline associated with worse verbal episodic memory at follow-up	Age at T2 Sex/gender Time between time points Peripheral biomarkers of neuropathology and inflammation	Low	Higher AgeAccelGrim associated with higher externalizing factor scores (from CFA) Higher AgeAccelGrim associated with later increase in biomarkers of neuropathology and inflammation in blood
Phyo *et al.*, 2024 (84)	Horvath 1 Hannum PhenoAge GrimAge GrimAge2 (118) DunedinPACE (86)	Blood, EPIC	CS and LONGL (each year up to 9 y, mean: ∼7 y)	Subsample from ASPREE study, Australia, almost only White Australian	560 (51%)	75 (4, ≥70)	General cognition (ModifiedMMSE) Verbal episodic memory (Delayed Recall Task from HVLT-R) Verbal fluency (Controlled Oral Word Association Test, single letter F version) Processing speed (Symbol Digit Modalities Test)	Composite scores of overall cognitive performance (all 4 tests), executive function/processing speed, and verbal episodic memory and separate analysis for each test; multivariate linear mixed-effects regression models	Females: Higher DunedinPACE associated with worse verbal fluency, composite overall cognitive performance and composite executive function/processing speed at baseline (nominally with processing speed) Higher AgeAccelGrim2 associated with worse processing speed at (baseline nominally for AgeAccelGrim) Higher AgeAccelGrim/AgeAccelGrim2 nominally associated with worse composite executive function/processing speed at baseline Higher AgeAccelHannum nominally associated with worse verbal episodic memory and composite overall cognitive performance at baseline Higher AgeAccelGrim2 nominally associated with worse verbal episodic memory over time Higher AgeAccelPheno nominally associated with worse composite verbal episodic memory over time Males: Higher DunedinPACE nominally associated with worse general cognition at baseline	Age Education SES Smoking status Number of chronic conditions Cell type composition	Low	Analysis performed in a sex-stratified manner Continuous AgeAccel considered, but data also available for categorical and PC-trained versions (114) Higher AgeAccelGrim2 associated with increased risk of Dementia after 7 y in males (nominal for AgeAccelGrim)
Nguyen *et al.*, 2024 (24)	Horvath Hannum PhenoAge GrimAge2 GrimAge2 components DunedinPACE	Blood, 450K	LONGL (each year up to 13 y, mean: 8–9 y depending on study)	Subsamples from WHIMS -ECHO, and WHIMS-YA, United States, mixed ethnicities (majority White, 82%–84% depending on study)	WHIMS: 758 (100%) WHIMS-ECHO/WHIMS-Y: 376 (100%)	WHIMS: 71 (4, ≥65)^f^ WHIMS-ECHO/WHIMS-Y: 68 (7)^f^	General cognition (Modified MMS at WHIMS, modified TICS at WHIMS-ECHO/WHIMS-Y)	General cognition; multivariate linear mixed-effects regression models	Higher DunedinPACE associated with faster decline in general cognition over time	Age Education Ethnicity Smoking status Alcohol consumption status BMI Comorbidities (hypertension, diabetes, coronary heart disease) Study Hormone therapy treatment group Cognitive function at baseline Quadratic time effect Cell type composition (for IEAA)	Low	AgeAccel for Horvath and Hannum clocks estimated using IEAA and EEAA (119), respectively Higher DunedinPACE associated with faster decline in general cognition only among *APOE* e4 carriers Consistent results when accounting for death during follow-up
Graves *et al.*, 2024 (83)	GrimAge	Blood, EPIC	CS	Subsample from VCAP, United States, mixed ethnicities (majority White, 90%)	103 (71%)	69 (6,59–81)	Processing speed (Digit Symbol Substitution from WAIS-III, Pattern Comparison, Letter Comparison) Episodic memory (Word Recall and Logical Memory from WMS-III, Paired Associates) Visuospatial abilities (Spatial Relations, Paper Folding, Form Boards) Reasoning (Matrix Reasoning, Shipley Abstraction, Letter Sets) Crystallized intelligence (Vocabulary Tests from WAIS-III)	Latent variables (from bootEGA) representing processing speed, episodic memory, reasoning/visuospatial abilities, and crystallized intelligence; multivariate hierarchical Bayesian models	Higher AgeAccelGrim associated with worse processing speed, episodic memory, reasoning/visuospatial abilities, crystallized intelligence	Age Sex/gender Time points White blood cell count	Low	Mean of 3 assessment sessions taken for cognitive measures AgeAccelPheno/AgeAccelHorvath not included because specific multivariate results were not reported −1 SD increase in AgeAccelGrim resulted in ∼1/4–1/3 SD decrease across domains Similar resting-state functional connectivity profiles in brain predict AgeAccelGrim and episodic memory
Chen *et al.*, 2024 (25)	Horvath 1 Hannum PhenoAge GrimAge DunedinPACE	Blood, EPIC	CS and LONGL (∼35 y)	Subsamples from CHDS and ∼35 y later CHDS DISPAR Aging, United States, mixed ethnicities (majority non-Hispanic White, 57%)	359 (47%)	Childhood: 10 (1) Adolescence: 17 (1) Midlife: 49 (1)	Childhood: Crystallized intelligence (Peabody Picture Vocabulary Test 9) Fluid intelligence (Raven Colored Progressive Matrices 9) Adolescence: Crystallized intelligence (Peabody Picture Vocabulary Test 15) Midlife: Crystallized intelligence (WTAR) Fluid intelligence (Verbal Fluency, Digit Symbol from WAIS-R)	Crystallized intelligence and fluid intelligence in childhood, adolescence (only crystallized intelligence) and midlife; multivariate linear regression models	Higher AgeAccelPheno and DunedinPACE associated with worse crystallized intelligence in midlife Higher AgeAccelGrim/AgeAccelPheno/DunedinPACE associated with worse fluid intelligence in midlife No association between all AgeAccels in midlife and childhood cognitive function Higher AgeAccelGrim in midlife associated with worse crystallized intelligence in adolescence (nominally for DunedinPACE)	Age Sex/gender Ethnicity School grade level Household smoking status Cell type composition SES Additional variables in midlife analysis: Alcohol use Cigarettes use BMI Cognitive function in childhood and adolescence	Low	DNAm assessed in midlife Differences observed in imputed data
Vyas *et al.*, 2023 (81)	PhenoAge GrimAge DNAmTL	Blood, EPIC	CS and LONGL (∼2 y)	Subsample from VITAL-DEP study, United States, mixed ethnicities (majority non-Hispanic White, 87%)	45 (49%)	70 (6, ≥60)	General cognition (Modified MMSE) Verbal episodic memory (Immediate and Delayed Verbal Memory, EBMT) Executive function/attention (Category Fluency for Animals and Vegetables, TMT-A/B)	Global cognitive score (from all tests), general cognition and domain-specific cognitive scores for verbal episodic memory and executive function/attention; partial Spearman rank correlations at baseline and multivariate linear regression models for LONGL analysis	Higher AgeAccelGrim associated with worse global cognitive score at baseline (nominally with executive function/attention) Higher increase in DNAmGrimAge associated with faster decline in global cognitive score over time Higher increase in DNAmTL associated with better global cognition score over time Higher increase in DNAmPhenoAge nominally associated with faster decline in verbal episodic memory and executive function/attention over time Higher increase in DNAmGrimAge nominally associated with faster decline in executive function/attention over time Higher increase in DNAmTL nominally associated with better verbal episodic memory over time	Age Sex/gender Cell type composition BMI Smoking status Charlson-Deyo comorbidity index	Low	Participants with MCI and CN participants included ΔCognitive performance (at follow-up − at baseline) used as outcome and ΔDNAm as exposure in LONGL analysis
Stephan *et al.*, 2023 (80)	GrimAge	Blood, EPIC	LONGL (∼2 y)	Subsample from HRS and HRS VBS, United States, mixed ethnicities	2423 (NA for specific analysis)	NA for specific analysis	Verbal episodic memory (Immediate and Delayed Recall Task)	Verbal episodic memory (sum of all tests); multivariate linear regression models	Higher AgeAccelGrim associated with worse verbal episodic memory ∼2 y later	Age Sex/gender Education Race Ethnicity Wave of subjective age assessment Other biomarkers	Low	Only association effects of mediator epigenetic aging considered (study focused on mediation analysis for subjective age on cognition)
Robinson *et al.*, 2023 (79)	Horvath 2	Blood, 450K	CS	Subsample from HELIX study, multiple countries (United Kingdom, France, Spain, Norway, Lithuania, and Greece), mixed ethnicities (majority White, 89%)	802–1052 (NA for specific analysis)	NA for specific analysis	Fluid intelligence (Raven Colored Progressive Matrices Test, computer-based) Attention (Attention Network Test, computer-based) Working memory (n-Back Task, computer-based)	Fluid intelligence, attention, working memory; multivariate linear regression models	No association between DeltaAgeHorvath2 and all cognitive tests	Age Sex/gender Ethnicity Study center Cell type composition Health risk factors (family affluence, social capital, birth weight, maternal active smoking, child passive smoking)	Low	DeltaAge defined as DNAmAge − chronological age
O’Shea and Galvin, 2023 (78)	GrimAge PhenoAge	Blood, EPIC	CS	Subsamples from HRS: VBS and HCAP, United States, mixed ethnicities (majority White, 76%)	1771 (57%)	75 (7,64–98)	Verbal episodic memory and learning (Immediate, Delayed Recall and Recognition Task from CERAD, Immediate and Delayed Recall from Logical Memory from WMS-III, Brave Man Story from EBMT, Logical Memory Story)	Composite scores of verbal episodic memory and learning; ANCOVA	Higher DNAmGrimAge rate group associated with worse verbal episodic memory and learning (verbal learning only nominally in men)	Age Sex/gender Ethnicity Education White blood cell count rates (from GrimAge) Cell type composition *APOE* ε4 carrier status Depressive symptoms	Low	Analysis performed in sex-stratified manner using categories defined by AgeAccel: slow, average, and fast agers Based on 1 SD above/below sex-specific mean age rate Slow rates of GrimAge associated with better memory performance in female *APOE* ε4 carriers
Mareckova *et al.*, 2023 (77)	Horvath 1	Buccal, EPIC	CS	Subsamples from ELSPAC and follow-up studies: Biomarkers and VULDE, HBA, Czech Republic, White	261 (48%)	29 (1)	Performance IQ (picture completion, Digit Symbol Coding, Matrix reasoning from WAIS-IV) Verbal IQ (Information, Arithmetic, Similarity, Digit Span from WAIS-IV)	IQ, performance IQ, verbal IQ; multivariate linear regression models	Higher EpiAGE associated with worse IQ in women (not in men) Sex-specific effects possibly driven by performance IQ (not verbal IQ)	Age Smoking frequency BMI Time point Proportion of epithelial cells	Low	EpiAGE calculated by regressing epigenetic age on age, time point, and proportion of epithelial cells EpiAGE associated with BrainAge
Lynch *et al.*, 2023 (76)	Cortical Clock (120)	Postmortem brain, 450K	CS	Subsamples from ROS or MAP, United States, mixed ethnicities (non-Latino White)	258 (63%)	NA for specific analysis	Verbal episodic memory (Logical Memory Ia and IIa, Immediate and Delayed Story Recall, Word List Memory, Word List Recall, Word List Recognition) Semantic memory (Boston Naming Test, Category Fluency Fruits and Animals, Extended Range Vocabulary, NART) Working memory (DSF, DSB, Digit Ordering, Alpha Span) Processing speed (Symbol Digit, Number Comparison) Visuospatial abilities (Judgment of Line Orientation, Standard Progressive Matrices)	Global cognition (averaged from all tests) and cognitive decline (person-specific rate of change in global cognition over time estimated from multivariate linear mixed-effects regression models controlling for age at baseline, sex/gender, and years of education); multivariate linear regression models	Higher DNAmAgeCorticalClock at death associated with worse prior global cognition Higher AgeAccelCorticalClock at death (binary as accelerated vs. not accelerated) associated with worse prior global cognition and cognitive decline	Age at death Sex/gender Education Neuronal cell type proportions	Low	Tissue originated from DLPFC Due to statistical design of models, findings were considered by authors as CS Higher DNAmCorticalClock associated with more pathological measures of AD and Lewy bodies Multimodal AgeAccel (cortical clock and mitochondrial DNA copy number) showed greater effect size than single measures
Lynch *et al.*, 2023 (75)	Horvath 1 Horvath 2 Hannum PhenoAge GrimAge DunedinPoAm	Blood, EPIC	CS	Subsamples from HRS, United States, mixed ethnicities (majority White, 77%)	1814 (59%)	70 (10,50–98)	Verbal episodic memory (Immediate and Delayed Recall) Working memory (Serial Sevens Subtraction Test) Processing speed (Counting Backwards Test) All measures from modified TICS	General cognition (composite score) and separate analysis for each test of the following cognitive domains: verbal episodic memory and processing speed; multivariate linear regression models	Higher AgeAccelGrim associated with worse general cognition and verbal episodic memory	Sex/gender Education Birth cohort Ethnicity BMI Current smoking status Depressive symptoms Self-rated health Social participation *APOE* ε4 status	Low	Only association effects of mediator epigenetic aging considered (because study focused on mediation analysis for loneliness on cognition)
Li *et al.*, 2023 (74)	Horvath 1 Hannum PhenoAge	Blood, 450K	CS	Patients with SCZ from Henan Mental Hospital and HC, China, ethnicity not specified	76 (34%)	25 (5)	Verbal fluency (Verbal Fluency Test) Executive function (TMT-A/B, Stroop Color Word and Interference Tests, Wisconsin Card Sorting Test) Working memory (DSF/DSB)	Separate analysis for each test of following cognitive domains: verbal fluency, executive function, working memory; multivariate linear regression models	No association between all AgeAccels and all cognitive tests	Age Sex/gender Tobacco use	Low	38 drug-naïve first-episode SCZ patients and 38 HC participants included Only CS data considered (because study focused on associations of AgeAccel with cognition after 8-week risperidone treatment as intervention) Lower AgeAccelHorvath present in drug-naïve first-episode patients with SCZ compared with HC participants In patients, increase in AgeAccelHannum and AgeAccelPheno after 8-week risperidone treatment (also controlling for illness duration)
Jokai *et al.*, 2023 (73)	PhenoAge GrimAge DNAmFitAge (121)	Blood, EPIC	CS	Healthy and life-long athletes, Hungary, mixed ethnicities (majority White)	294 (52%)	33–88 (mean and SD NA for specific analysis)	Working memory (Digit Span Test)	Working memory; Pearson, Spearman, and Kendall correlation analyses	Higher AgeAccelFitAge associated with worse working memory	Age	Moderate	AgeAccelFitAge associated with regular exercise and multiple physiological fitness measures
Heany *et al.*, 2023 (72)	Horvath 1 Hannum	Blood, EPIC	CS	Subsample from CTAAC study, South Africa, Black African	35 (50%)^f^	T1: 11 (1)^f^ T2: 14 (1)^f^	General intellectual functioning (Vocabulary, Similarities, Block Design, and Matrix Reasoning from WASI) Sustained attention (Children’s Colour Trails Test 1 and 2) Working memory (DSB from WISC-IV, Children’s Colour Trails Test 1 and 2, Naming, Inhibition and Switching from A NEPSY-II) Visual memory (Immediate and Delayed Recall from Rey Complex Figure Test) Verbal episodic memory (Immediate, Delayed Recall and Recognition from HVLT-R) Visuospatial abilities (Rey Osterich Complex Figure Test) Linguistic abilities (Boston Naming Test-South African-Short Form, Category and Phonemic Fluency) Processing speed (Symbol Search and Coding from WISC -IV, Naming, Inhibition and Switching from form A NEPSY-II) Executive function (Naming, Inhibition and Switching from form A NEPSY-II, Similarities and Matrix Reasoning from WASI, Children’s Colour Trails Test 1 and 2, Verbal Fluency Category and Phonetic)	Separate analysis for each of following cognitive domains (sum score of tests): general intellectual functioning, sustained attention, working memory, visual memory, visuospatial abilities, verbal episodic memory, linguistic abilities, processing speed, executive function; correlation analysis	Higher AAD nominally associated with worse executive function and processing speed	Age	Low	Only HC participants considered (because study focused on HIV-positive adolescents) AgeAccel for Horvath and Hannum clocks estimated using AAD and EEAA, respectively Relevant analysis for HC participants and cognitive tests available only at follow-up, therefore considered as CS Motor coordination also evaluated but omitted here due to lack of specific cognitive domain assessment
Felt *et al.*, 2023 (71)	Horvath 1 Hannum PhenoAge GrimAge DunedinPoAm	Blood, EPIC	CS	Subsamples from BeCOME study and FGDS, Germany and United States, mixed ethnicities (majority White, BeCOME: 89%, FGDS: 52%)	BeCOME: 313 (64%) FGDS: 86 (100%)	BeCOME: 35 (12,18–66) FGDS: 37 (4)	Verbal episodic memory (Delayed-Recall Task from Materialien und Normwerte für die neuropsychologische Diagnostik) Verbal fluency (Word Fluency Task from Materialien und Normwerte für die neuropsychologische Diagnostik) Linguistic abilities (Multiple-Choice Vocabulary Intelligence Test) Executive function (Cognitive Flexibility Task and Go/No-Go Task from Test for Attentional Performance) Sustained attention (d2 Test)	Latent variables (from CFA) representing general cognitive abilities and speeded cognitive abilities; structural equation models	Higher AgeAccelHorvath associated with worse processing speed Higher DunedinPoAm nominally associated with worse general cognition	Age Sex/gender Genetic ancestry (PCs) Polygenic score for educational attainment Childhood maltreatment status Lifetime trauma Psychiatric burden Cell type composition	Low	Only BeCOME considered (analysis also performed in FGDS but omitted due to inclusion criteria)
Faul *et al.*, 2023 (70)	Horvath 1 Hannum PhenoAge GrimAge DunedinPACE	Blood, EPIC	CS	Subsamples from HRS: VBS and HCAP and subsample of HCAP nonparticipants, United States, mixed ethnicities (majority White, 81%)	3581 (55%)	68 (9)	Verbal episodic memory (Immediate and Delayed Recall Test) Working memory (Serial Sevens Subtraction Test) Processing speed (Counting Backwards Test) All measures from modified TICS	Cognitive function (composite score); ordinary least squares regression models	Higher AgeAccelPheno/AgeAccelGrim/DunedinPACE (also as PC-trained versions) associated with worse cognitive function	Age Sex/gender Ethnicity Education Smoking status Alcohol use Obesity Depression Childhood SES Childhood financial hardship Cell type composition	Low	AgeAccel estimated as original and PC-trained versions (114) Prior depression associated with higher AgeAccelHannum/AgeAccelGrim (original versions)/AgeAccel of all PC-trained Clocks/DunedinPACE AgeAccelPheno/AgeAccelGrim/DunedinPACE associated with health outcomes after 2 y (daily living difficulties and chronic conditions)
Arpawong *et al.*, 2023 (69)	GrimAge	Blood, EPIC	CS	Subsample from HRS, United States, European ancestry	2311 (56%)	72 (10,50–98)	Verbal episodic memory (Immediate and Delayed Recall Test) Working memory (Serial Sevens Subtraction Test) Processing speed (Counting Backwards Test) All measures from modified TICS	Cognitive function (composite score); structural equation models	No association between DNAmGrimAge and cognitive function	Age Sex/gender Smoking pack years Depressive symptoms BMI Years of education Household income	Low	Only association effects of mediator epigenetic aging considered (because study focused on mediation analysis for multiple variables on cognition) Higher DNAmGrimAge associated with higher polygenic score for ADHD (partial mediation via smoking, depressive symptoms, and education)
Zheng *et al.*, 2022 (68)	Horvath 1 Hannum PhenoAge GrimAge	Blood, EPIC	LONGL (5 and 15 y)	Two subsamples from CARDIA study, United States, mixed ethnicities (majority White, 60%)	890–932 (NA for specific analysis)	NA for specific analysis	Executive function (Stroop Color and Word Test) Verbal episodic memory (Delayed Recall from RAVLT) Processing speed (Digital Symbol Substitution Test from WAIS-III)	Separate analysis for each test of following domains: executive function, verbal episodic memory, processing speed/attention/working memory; multivariate linear regression models	Higher AgeAccelGrim associated with worse executive function, verbal episodic memory, and processing speed after 5 and 15 y	Age Sex/gender Ethnicity Study center Education Cell type composition (for IEAA)	Low	Two data collection time points were joined for both epigenetic age and cognitive function AgeAccel for Horvath and Hannum clocks estimated using IEAA and EEAA, respectively Combination of epigenetic aging and brain aging presented highest prediction accuracy for global cognitive function (from PCA) in the smaller subsample
Vetter *et al.*, 2022 (67)	Horvath 1 Hannum PhenoAge GrimAge 7-CpG clock (122)	Blood, EPIC (with exception of 7-CpG clock)	CS and LONGL (up to 10 y, mean: ∼7 y, only for 7-CpG clock)	Subsamples from GendAge study and BASE-II, Germany, ethnicity not specified	CS: 783–786 (NA for specific analysis) LONGL: 677 (NA for specific analysis)	NA for specific analysis	General cognition (MMSE)	General cognition; multivariate linear regression models	No association between all AgeAccels at T2 and general cognition at T2 No association between AgeAccel for 7-CpG clock at baseline and general cognition after up to 10y	Age Sex/gender White blood cell count Alcohol consumption Smoking behavior BMI Genetic ancestry (PCs) Morbidity index	Low	LONGL analysis available only for 7-CpG clock
Sugden *et al.*, 2022 (66)	Horvath 1 Hannum PhenoAge GrimAge DunedinPACE	Blood, 450K, EPIC	CS	Subsample from ADNI, United States, almost only White, 98%	649 (44%)	75 (8,55–96)	General cognition (Alzheimer’s Disease Assessment Scale-Cognitive Subscale, MMSE, MoCA) Verbal episodic memory (Immediate and Delayed Recall from RAVLT, Logical Memory Test from WMS-R) Executive function (TMT-B)	General cognition and separate analysis for each test of following domains: verbal episodic memory, executive function; multivariate linear regression models	Higher DunedinPACE/AgeAccelPheno associated with worse verbal episodic memory	Age Sex/gender Technical variation (plate number) Cell type composition	Low	Cohort included CN participants and participants with MCI and AD-dementia DunedinPACE also associated with clinical AD diagnosis Higher DunedinPACE associated with risk of developing dementia in additional LONGL analysis of Framingham Heart Study offspring cohort (*n* = 2264)
Sommerer *et al.*, 2022 (65)	Horvath 1	Blood, buccal, EPIC	CS and LONGL (mean: ∼5 y)	Subsamples from BASE-II, GendAge study, and BBHI, Germany and Spain, ethnicity not specified	CS: buccal, 1019; blood, 800 (50%) LONGL: buccal, 626; blood: 735 (50%–51%)	CS: buccal, 69 (11,30–90); blood, 76 (4,65–90) LONGL: 70 (4,61–85)	Episodic memory (BASE-II/GendAge: Verbal Learning and Memory Test, Face-Profession Task, Scene Encoding Task, Object Location Task; BBHI: Face-Name Associative Memory Exam, S-FNAME, RAVLT)	First PC (from PCA) representing episodic memory for CS analysis, first PC (from PCA) representing annual percentage of change for each test of episodic memory for LONGL analysis; multivariate linear regression models	No association between AgeAccelHorvath (in both tissue types) and episodic memory at baseline and over time	Age Cell type composition Sex/gender PCs of technical variation (e.g., laboratory batch, array)	Low	Only CS data available for BBHI EWAS to identify differentially methylated probes for episodic memory function and analysis of polyepigenetic scores of cognitive abilities and AD also available
Segura *et al.*, 2022 (64)	Horvath 1 Hannum PhenoAge DNAmTL	Blood, EPIC	CS	Subsample from clinical and neurobiological determinants of second episodes of SCZ. Longitudinal study of first episode of psychosis (2EPs Project), Spain, mixed ethnicities (majority White, >86%)	91 (Nonrelapse: 22%) (Relapse: 36%)	Nonrelapse: 27 (6) Relapse: 26 (6)	Verbal episodic memory (Short and Long Delay Free Recall, Short and Long Delay Cued Recall, Immediate Recall from CVLT) Visual memory (Immediate and Delayed Recall from Brief Visuospatial Memory Test-Revised) Executive function (Tower of London) Sustained attention (Continuous Performance Test–II) Working memory (Digits, Letter Number Sequencing from WAIS-III) Verbal fluency (Semantic: Animals and Phonemic: F-A-S) Processing speed (TMT-A, Digit Symbol from WAIS-III)	PCs (from PCA) representing verbal episodic memory, visual memory, executive function, sustained attention, working memory, verbal fluency, processing speed; partial correlation analyses	Higher EEAA/DNAmTL nominally associated with worse working memory and verbal fluency	Age Cell type composition (for IEAA)	Low	42 relapsed and 49 nonrelapsed participants (during follow-up of 3 y) AgeAccel for all epigenetic clocks estimated as IEAA and EEAA versions Higher AgeAccelDNAmTL in relapsed patients with SCZ over 3 y
Reed *et al.*, 2022 (63)	Horvath 1 Hannum PhenoAge GrimAge DunedinPoAm DunedinPACE	Blood, EPIC	LONGL (mean: 16 y, range: 15–17 y)	Subsample from AHAB-1 study, United States, mixed ethnicities (majority White, 92%)	48 (56%)	T1: 45 (6) T2: 61 (6)	Executive function (TMT-A. minus B, Stroop Color-Word Test) Processing speed (TMT-A, Stroop Color-Word test) Nonverbal reasoning (matrix reasoning from WAIS) Working memory (DSF/DSB from WAIS-III) Sustained attention (Digit Vigilance)	Separate analysis for each test of following cognitive domains: executive function, processing speed, nonverbal reasoning, working memory, sustained attention; multilevel models	Higher AgeAccelPC-GrimAge/DunedinPoAm/DunedinPACE associated with worse executive function over time Higher DunedinPACE nominally associated with slower processing speed and nonverbal reasoning over time	Age Sex/gender Cell type composition	Low	AgeAccel estimated also as PC clocks (114) Due to statistical design of models, findings can be considered as AgeAccel Higher AgeAccelPC-GrimAge/DunedinPoAm/DunedinPACE in decliners over time (nominal, effect remained over time) when comparing decliners (with most cognitive decline over time) and maintainers (matched control participants)
Pérez *et al.*, 2022 (62)	Horvath1 Hannum PhenoAge GrimAge DNAmTL	PBMCs, EPIC	LONGL (∼4 y)	Subsample from Vallecas Project, Spain, White	34 (CN: 53%) (DEM: 82%)	CN: 76 (3) DEM: 77 (4)	General cognition (MMSE)	General cognition; linear regression models (all participants from both time points included)	No association between all AgeAccels and general cognition	Age Sex/gender (only for GrimAge)	Low	17 pairs of age-matched CN participants were divided into CN and DEM groups based on whether DEM was present at follow-up AgeAccel did not differ between DEM and CN groups at follow-up EWAS of DEM vs. CN also available
Milicic *et al.*, 2022 (61)	Horvath1 Hannum PhenoAge Zhang 1 (123) Zhang 2 (123)	Blood, EPIC	CS and LONGL (mean: ∼6 y, up to 11 y)	Subsample from AIBL study and ADNI, multiple countries (Australia, United States, and Canada), mixed ethnicities (majority White)	AIBL: NA for specific analysis (53%)^f^ ADNI: 469 (47%)^f^	AIBL: NA for specific analysis ADNI: 74 (8)^f^	General cognition (MMSE) Verbal episodic memory (Free and Cued Selective Reminding Test), Logical Memory IIa from WMS) Processing speed (DSST from WAIS-R)	PACC; multivariate linear regression models	No association between all AgeAccels and PACC scores at baseline or over time (only tested in AIBL)	Age Sex/gender Years of education Smoking status *APOE* ε4 carrier status	Low	Only CN group considered without stratification by amyloid-β status (PET) No association between AgeAccel and amyloid-β burden (PET) at baseline or over time Higher AgeAccelHannum/AgeAccelPheno associated with reduced hippocampal volume at baseline (only in AIBL cohort, nominally for AgeAccelHannum over time)
Lima *et al.*, 2022 (60)	GrimAge	Blood, EPIC	CS	Patients with BPD and HC participants from UTHealth Center of Excellence on Mood Disorders, United States, mixed ethnicities (BPD: majority White, 41%; HC: majority African American, 44%)	186 (BPD: 72%)^f^ (HC: 68%)	BPD: 37 (11)^f^ HC: 36 (10)	Verbal episodic memory (short-term and delayed affective and nonaffective memory) Executive function (Inhibition, Problem solving) Verbal fluency All measures from Brief Assessment of Cognition in Affective Disorders	Separate analysis for each test of the following cognitive domains: verbal episodic memory, executive function, verbal fluency; multivariate linear regression models	BPD: higher AgeAccelGrim nominally associated with worse verbal episodic memory and executive function HC: higher AgeAccelGrim nominally associated with worse executive function	Age Cell type composition	Low	136 patients with BPD and 50 age/sex/ethnicity-matched HC participants Analysis performed separately in patients with BPD and HC participants Analysis of cognitive function performed only for AgeAccelGrim (associated with BPD) Affective and nonaffective memory tests grouped to verbal episodic memory Token motor speed also evaluated but omitted due to lack of specific cognitive domain assessment
Krivonosov *et al.*, 2022 (59)	Horvath 1 Hannum PhenoAge GrimAge	Blood, EPIC	CS	Volunteers (additional details not available)	47 (57%)	NA (25–85)	Executive function (Sensorimotor tests with arithmetic calculation and identification of reversed letters, web-based platform)	Separate analysis for each test of executive function; Pearson correlation analysis	Higher DNAmAgeHorvath/DNAmAgeHannum/DNAmPhenoAge/DNAmGrimAge associated with worse executive function	None	Moderate	Campimetry test also evaluated but omitted here due to lack of specific cognitive domain assessment
Belsky *et al.*, 2022 (86)	Horvath 1 Hannum PhenoAge GrimAge DunedinPoAm DunedinPACE	Blood, EPIC	CS and LONGL (∼38 y)	Subsample from Dunedin Multidisciplinary Health and Development Study, New Zealand, mixed ethnicities (majority White)	814 (NA for specific analysis)	NA for specific analysis	Nonverbal reasoning Working memory Processing speed All measures from WAIS-IV or WISC-R	Separate analysis for each of the following cognitive domains: nonverbal reasoning, working memory, processing speed, and cognitive decline (IQ change since childhood) (WAIS-IV at 45 y − average of WISC-R at 7, 9, 11, and 13 y); multivariate linear regression models	Higher DunedinPACE/DunedinPoAm/DNAmGrimAge at T2 associated with worse nonverbal reasoning, working memory, and processing speed at T2 Higher DNAmAgeHorvath at T2 nominally associated with worse processing speed at T2 Higher DNAmAgeHannum/DNAmPhenoAge at T2 nominally associated with worse nonverbal reasoning at T2 Higher DNAmGrimAge associated with worse prior cognitive decline over time	Sex Smoking pack years White blood cell count	Low	Results considered only if survived all corrections for covariates (separate models)
Vaccarino *et al.*, 2021 (58)	Horvath1 Hannum PhenoAge GrimAge	PBMCs, EPIC	CS and LONGL (mean: ∼12 y, range: 7–16 y)	Subsample from ETS, United States, mixed ethnicities (majority White, 97%)	CS: 266 [133 twin pairs] (0%) LONGL: 114 [57 twin pairs] (0%)	56 (3)	Executive function (TMT-A/B) Verbal episodic memory (Immediate and Delayed Recall from Logical Memory from WMS-R)	Composite scores representing executive function and verbal episodic memory; multivariate linear mixed-effects regression models	No association between all AgeAccels and executive function or verbal episodic memory at baseline Higher AgeAccelHorvath/IEAA associated with worse executive function and verbal episodic memory over time	Years of education Smoking status BMI Hypertension Alcohol abuse Diagnosis (MDD, PTSD) Cell type composition (for IEAA) Baseline cognitive function (only in LONGL analysis)	Low	Twins discordant for MDD or PTSD and HC participants included AgeAccel for Horvath and Hannum clocks estimated also using IEAA and EEAA, respectively Analysis performed in within-twin pair manner Missing cognitive values were imputed
Shiau *et al.*, 2021 (56)	Horvath1 Hannum GrimAge PhenoAge DNAmTL	Blood, EPIC	CS	Sample from CUIMC, United States, African American (5%–7% also identified as Hispanic/Latino)	38 (42%)	66 (5,60–78)	General cognitive function (MoCA) Executive function/attention (DCCS, Flanker Inhibitory Control and Attention Test) Working memory (List Sorting Working Memory Test) Processing speed (Pattern Comparison Processing Speed Test) Linguistic abilities (Oral Reading Recognition Test) All measures (beyond general cognitive function) from National Institutes of Health Toolbox Cognition Battery	Separate analysis for general cognition and each test of the following cognitive domains: executive function/attention, working memory, processing speed, linguistic abilities; Pearson correlation analysis	No association between all AgeAccels and all cognitive tests	Age Cell type composition (for IEAA)	Low	Only HC participants considered (because study focused on comparison with HIV-positive participants) AgeAccel for Horvath and Hannum clocks estimated using AgeAccel/IEAA and EEAA, respectively
Shiau *et al.*, 2021 (57)	Horvath1 Hannum	Blood, EPIC	CS	Sample from CUIMC, United States, African American (10% also identified as Hispanic/Latino)	30 (57%)	28 (4,21–35)	Executive function/attention (DCCS, Flanker Inhibitory Control and Attention Test) Working memory (List Sorting Working Memory Test) Processing speed (Pattern Comparison Processing Speed Test) Linguistic abilities (Oral Reading Recognition Test) All measures from National Institutes of Health Toolbox Cognition Battery	Separate analysis for each of the following cognitive domains: executive function/attention, working memory, processing speed, linguistic abilities; Pearson correlation analysis	Higher IEAA nominally associated with worse linguistic abilities	Age Cell type composition (for IEAA)	Low	Only HC participants considered (because study focused on comparison with HIV-positive participants) AgeAccel for Horvath and Hannum clocks estimated using AgeAccel/IEAA and EEAA, respectively
Park *et al.*, 2021 (55)	Horvath1 Hannum	Blood, EPIC	CS	Subsample from KFACS, Korea, ethnicity not specified	29 (55%)	76 (4)	General cognition (MMSE) Verbal episodic memory (Immediate, Delayed Recall and Recognition Task) Working memory (DSF/DSB) Executive function (Frontal Assessment Battery) All measures from Korean version of CERAD (CERAD-K)	Separate analysis for general cognition and each of the following cognitive domains: verbal episodic memory, working memory, executive function; Pearson correlation analysis	Higher AgeAccelHorvath nominally associated with worse executive function Higher EEAA nominally associated with worse verbal episodic memory	Age Cell type composition (for IEAA)	Low	AgeAccel for Horvath and Hannum clocks estimated using AgeAccel/IEAA and EEAA, respectively
McCrory *et al.*, 2021 (54)	Horvath1 Hannum PhenoAge GrimAge	Blood, EPIC	CS	Subsample of TILDA, Ireland, mixed ethnicities (majority White)	460–488 (50%) f	62 (8,50–90)^f^	General cognition (MoCA, MMSE) Sustained attention (Sustained Attention Reaction Time) Processing speed (2-Choice Reaction Time Task)	Separate analysis for general cognition and each of the following cognitive domains: processing speed, sustained attention; multivariate negative binomial regression analysis (multivariate ordinary least squares regression analysis for processing speed)	No association between all AgeAccels and all cognitive tests	Age Sex/gender Cell type composition Life course SES trajectory Smoking status Hazardous alcohol consumption Physical activity BMI	Low	
Hillary *et al.*, 2021 (53)	GrimAge	Blood, 450K	CS and LONGL (up to 9 y)	Subsample of LBC1936, United Kingdom, White	666 (48%)	CS: 73 (1) LONGL: 70 (1)	General cognition (MMSE) Processing speed (Digit Symbol Coding and Symbol Search from WAIS-III, Simple and Four Choice Reaction Time) Nonverbal reasoning (Matrix Reasoning from WAIS-III) Visuospatial abilities (Block Design from WAIS-III) Working memory (Letter Number Sequencing and Backward Digit Span from WAIS-III, Spatial Span from WMS-III) Crystallized intelligence (WTAR, NART) Verbal episodic memory (Logical Memory I, II and Verbal Paired Associates from WMS-III) Verbal fluency (Naming Letters C, F, and L)	First PC (from PCA) representing general cognitive ability score (nonverbal reasoning, working memory, processing speed, visuospatial abilities), general cognition, and each test of the following cognitive domains: processing speed, nonverbal reasoning, visuospatial abilities, working memory, crystallized intelligence, verbal episodic memory, verbal fluency; multivariate linear regression models at baseline, multivariate linear mixed-effects regression models for LONGL analysis	Higher AgeAccelGrim associated with worse general cognitive ability score, processing speed, and nonverbal reasoning No association between AgeAccelGrim and cognitive decline after 9y	Age Sex/gender IQ at age 11	Low	LONGL analysis performed only for general cognitive ability score Higher AgeAccelGrim associated with lower ratios of white matter volume, brain volume, and gray matter volume to intracranial volume and positively associated with ratio of white matter hyperintensities volume to intracranial volume No association between AgeAccelGrim and *APOE* ε4 carrier status Visual discrimination also evaluated but omitted here due to lack of specific cognitive domain assessment
Grodstein *et al.*, 2021 (52)	Horvath1 Hannum PhenoAge GrimAge Cortical clock	Postmortem brain, 450K	LONGL (every year until death)	Subsamples from ROS or MAP, United States, mixed ethnicities (majority White, 95%)	633–680 (64%)	88 (7)	Verbal episodic memory Semantic memory Working memory Processing speed Visuospatial abilities [see details regarding specific cognitive tests in Lynch *et al.*, 2023 (76) above]	Global cognition (averaged from all tests) and each of the following cognitive domains: verbal episodic memory, semantic memory, working memory, processing speed, visuospatial abilities; multivariate linear mixed-effects regression models	Higher AgeAccelGrim at death associated with lower odds of dementia and better prior cognitive decline in global cognition and working memory over time Higher DNAmAgeCortical at death associated with worse prior cognitive decline in global cognition, working memory, and visuospatial abilities over time	Age at death Sex/gender Education Depressive symptoms at baseline Neuronal cell type proportions (only for cortical clock)	Low	Tissue originated from the DLPFC Higher DNAmAgeCorticalDNAmAgeHannum/DNAmAgeHorvath/DNAmAgePheno and DNAmAgeCortical associated with postmortem AD pathology Higher AgeAccelCortical associated with greater odds of AD
Wiesman *et al.*, 2020 (51)	Horvath 1 Hannum	Blood, 450K	CS	Subsample from Magnetoencephalography and MRI Markers of HIV-Associated Neurocognitive Disorders Across the Lifespan, United States, ethnicity not specified	68 (49%)	45 (15,22–72)	Processing speed (TMT-A, Symbol Search) Executive function (Digit Symbol, Stroop Color-Word Interference Test, TMT-B) Verbal fluency Linguistic abilities (semantic Fluency)	Composite scores representing the following cognitive domains: processing speed, attention, executive function; Pearson correlation analysis	No association between averaged AgeAccel and processing speed/attention/executive function	Age Cell type composition	Low	Averaged AgeAccel (termed DeltaAge) calculated using average of DNAmHorvath and DNAmHannum regressed on chronological age Motor coordination (grooved pegboard) also evaluated but omitted due to lack of specific cognitive domain assessment Cognitive tests categorized differently in analysis Averaged DNAmAge predicted selective attention–related gamma activity in the anterior cingulate cortex
Maddock *et al.*, 2020 (50)	Horvath 1 Hannum PhenoAge GrimAge	Blood, EPIC	CS and LONGL (7–16 y)	Subsamples from Medical Research Council NSHD and NCDS, United Kingdom, White	NSHD: 1322–1348 [LONGL: 1317–1327] (T1: 52%)^f^ (T2: NA for specific analysis) NCDS: 238–240 (53%)^f^	NSHD T1: 53 (0.2)^f^ NSHD T2: NA for specific analysis NCDS: 45 (0.4)^f^	Verbal episodic memory (Immediate Recall) Processing speed (Letter Cancellation Test)	Separate analysis for each test of the following cognitive domains: verbal episodic memory, processing speed, and change in tests over time; multivariate linear regression models, meta-analytic approach at baseline, multivariate linear mixed-effects regression models for LONGL analysis	Higher AgeAccelPheno associated with worse processing speed at baseline Higher AgeAccelGrim associated with worse verbal episodic memory at baseline Higher AgeAccelGrim associated with worse verbal episodic memory and processing speed over time Higher AgeAccelPheno nominally associated with worse verbal episodic memory and processing speed over time Higher AgeAccelHannum nominally associated with worse verbal episodic memory over time	Age Sex/gender BMI Height Smoking status Social class	Low	Time differences present between DNAm measurement and cognitive assessment at baseline: 5 y in SCDS and 5–9 y at NSHD T2
Li *et al.*, 2020 (49)	Horvath 1 Hannum PhenoAge GrimAge	Blood, 450K	LONGL (up to 28 y)	Subsample from SATSA, Sweden, White	288 (57%)	68 (9)	Crystallized intelligence (Information from Central Värnpliktsbyrån scales, Synonyms from Dureman–Sälde Battery, Analogies from Westrin Intelligence Test-III) Fluid intelligence (Figure Logic from Dureman–Sälde Battery, Koh’s Block Design, Card Rotations from Educational Testing Service) Working memory (Digit Span Test) Visual memory (Thurstone’s Picture Memory) Processing speed (Symbol Digit, Figure Identification from Dureman–Sälde Battery)	First PC (from PCA) representing general cognitive ability (all time points standardized to first assessment); repeated-measures correlation analysis	No association between all AgeAccels and general cognitive ability (considering changes over time)	Age Sex/gender Individual- and twin pair–related components	Low	AgeAccels calculated by regressing out effects of chronological age, sex, individual- and twin pair–related components Higher AgeAccel using all measurements associated with increased mortality risk beyond effects of sex, educational attainment, smoking status. and BMI over median follow-up of 15 y
Hoare *et al.*, 2020 (48)	Horvath 1 Hannum	Blood, EPIC	CS	Subsample from CTAAC study, South Africa, Black African	44 (55%)	11 (1,9–12)	General intellectual functioning Sustained attention Working memory Visual memory Verbal episodic memory Linguistic abilities Visuospatial abilities Processing speed Executive function [see details regarding specific cognitive tests in Heany *et al.*, 2023 (72) above]	Separate analysis for each of the following cognitive domains (sum score of tests): general intellectual functioning, sustained attention, working memory, visual memory, visuospatial abilities, verbal episodic memory, linguistic abilities, processing speed, executive function; Pearson correlation analysis	Higher EEAA associated with worse visual memory and visuospatial abilities	Age Sex/gender	Low	AgeAccel for Hannum clock estimated using EEAA Motor coordination also evaluated but omitted due to lack of specific cognitive domain assessment
Hillary *et al.*, 2020 (85)	Horvath 1 Hannum PhenoAge GrimAge DunedinPoAm DNAmTL	Blood, EPIC	CS	Subsample from GS:SFHS, United Kingdom, mixed ethnicities (majority White)	Discovery: 4291–4324 (NA for specific analysis) Replication: 2504–2529 (61%)^f^	Discovery: NA for specific analysis Replication: 50 (13)^f^	Processing Speed (Wechsler DSST) Verbal episodic memory (Wechsler Logical Memory Test) Verbal fluency (Phonemic Verbal Fluency Test) Crystallized intelligence (Mill Hill Vocabulary Test)	First PC (from 2 separate PCAs) representing general cognitive ability score (processing speed, verbal episodic memory, verbal fluency, crystallized intelligence) and general fluid cognitive ability score (processing speed, verbal episodic memory, verbal fluency); multivariate linear regression models	No association between all AgeAccels and general cognitive ability or general fluid cognitive ability	Age Sex/gender Alcohol consumption BMI Deprivation Educational attainment Smoking pack years	Low	AgeAccel for Horvath and Hannum clocks estimated using AAD and EEAA, respectively Analysis split into discovery and replication cohort AgeAccelGrim associated with self-reported and diagnosed depression
Bressler *et al.*, 2020 (47)	Horvath 1 Hannum	Blood, ARIC: 450K GS:SFHS: EPIC	CS and LOGNL (∼6 y)	Two subsamples from ARIC study, United States, African Americans and European Americans, and subsample from GS:SFHS, United Kingdom, mixed ethnicities (majority White)	ARIC-1: 2135–2155 [LONGL: 1384–1401] (65%)^f^ ARIC-2: 705–708 (61%)^f^ GS:SFHS: 1670 (58%)	ARIC- 1: 56 (6,47–70)^f^ ARIC-2: 59 (5)^f^ GS:SFHS: 56 (6)	Processing speed (DSST from WAIS-R) Verbal fluency (Word Fluency Test) Verbal episodic memory (Delayed Word Recall Test, available only in ARIC)	Separate analysis for each test of the following domains: processing speed, verbal fluency, verbal episodic memory; inverse-variance weighted meta-analysis	Higher AgeAccelHannum associated with worse verbal fluency at baseline No association between AgeAccels and change in all cognitive tests over time (only in subsample of ARIC-1 study)	Age Sex/gender Years of education *APOE* Genotype BMI Smoking status Hypertension Diabetes Cell type composition Hypercholesterolemia (only in ARIC)	Low	Nominal associations for AgeAccelHannum with verbal fluency for separate analysis in ARIC-1 and GS:SFHS
Beydoun *et al.*, 2020 (46)	Horvath 1 Hannum	Blood, EPIC	CS and LOGNL (mean: 5 y)	Subsample from HANDLS study, United States, mixed ethnicities (majority African American, 51%)	147–156 (46%)	56 (0.1, >50)	General cognition (MMSE) Verbal episodic memory (List A and Delayed Free Recall from CVLT) Visuospatial abilities (Benton Visual Retention Test, Clock Drawing Test) Working memory (DSF/DSB) Attention/executive function (TMT-A/B, Brief Test of Attention) Verbal fluency (Animal Fluency Test)	Separate analysis for each test of following cognitive domains: general cognition, verbal episodic memory, visuospatial abilities, working memory, attention/executive function, verbal fluency; multivariate linear mixed-effects regression models	No association between AgeAccelHorvath/IEAA/EEAA and all cognitive tests at baseline Higher EEAA associated with faster decline in attention/executive function and visuospatial abilities in men over time	Age Sex/gender Ethnicity Marital status Educational level Poverty status Employment status Literacy BMI Dietary quality Chronic conditions Current drug use Current NSAID use Current smoking status Depressive symptoms Time between time points Inverse mills ratio Cell type composition (for IEAA)	Low	AgeAccel for Horvath and Hannum clocks estimated using AgeAccel/IEAA and EEAA, respectively
Vyas *et al.*, 2019 (45)	Horvath 1 Hannum	Blood, EPIC	CS	Subsample from VITAL-DEP study, United States, mixed ethnicities (majority White)	23 (Cases: 64%) (HC: 50%)	Cases: 67 (60–75) HC: 66 (56–73)^e^	General cognition (MMSE) Verbal episodic memory (Delayed Recall from EBMT)	General cognition, verbal episodic memory; Spearman rank correlations	Higher AgeAccelHorvath associated with worse general cognition	Age Cell type composition (for IEAA)	Moderate	11 psychiatric patients with various disorders and 12 HC participants included AgeAccel for Horvath and Hannum clocks estimated using AgeAccel/IEAA or EEAA, respectively
Stevenson *et al.*, 2019 (44)	PhenoAge	Blood, 450K	CS and LONGL (∼9 y)	Subsample from LBC1936, United Kingdom, White	838 (50%)^f^	70 (1)^f^	General cognition (MMSE) Processing speed (Digit Symbol Coding and Symbol Search from WAIS-III, Simple and Four-Choice Reaction Time) Nonverbal reasoning (Matrix Reasoning from WAIS-III) Visuospatial abilities (Block Design from WAIS-III) Working memory (Letter Number Sequencing and Backward Digit Span from WAIS-III, Spatial Span from WMS-III) Crystallized intelligence (WTAR, NART) Verbal episodic memory (Logical Memory I, II and Verbal Paired Associates from WMS-III)	Separate analysis for each test of following cognitive domains: general cognition, processing speed, nonverbal reasoning, visuospatial abilities, working memory, crystallized intelligence, verbal episodic memory; multivariate linear regression models at baseline, multivariate linear mixed-effects regression models for LONGL analysis	Higher AgeAccelPheno nominally associated with general cognition, crystallized intelligence, verbal episodic memory, verbal fluency, processing speed at baseline Higher AgeAccelPheno nominally associated with less decline in processing speed over time Higher AgeAccelPheno associated with lower IQ at age 11	Age Sex/gender IQ at age 11 (only in CS analysis)	Low	For better comparability, the following original cognitive categories were included in each overarching domain: spatial learning and memory (in working memory), verbal learning and memory (in verbal episodic memory), information processing (in processing speed) Visual discrimination also evaluated but omitted here due to lack of specific cognitive domain assessment
Cruz-Almeida *et al.*, 2019 (43)	Horvath 1	Blood, EPIC	CS	Subsample from NEPAL study, United States, mixed ethnicities (majority White)	23–28 (75%)^f^	71 (6, 60–83)^f^	Executive function (DCCS, Flanker Inhibitory Control and Attention) Nonverbal episodic memory (Picture Sequence Memory) Working memory (List Sorting) Processing speed (Pattern Comparison) Linguistic abilities (Oral Reading Recognition Test, Picture Vocabulary) All measures from NIH Toolbox Cognition Battery	Composite scores representing fluid intelligence (executive function, episodic memory, working memory, processing speed) and crystallized intelligence (linguistic abilities) and separate analysis for each test; partial correlation analysis	Higher DNAmAgeHorvath associated with worse fluid intelligence, nonverbal episodic memory, and working memory	Age Sex/gender Ethnicity	Low	No association between epigenetic aging and depressive symptoms For better comparability, the following original cognitive categories were included in each overarching domain: language and reading (in linguistic abilities)
Suarez *et al.*, 2018 (42)	Horvath 1	Blood, EPIC	CS	Subsample from GLAKU, Finland, ethnicity not specified	221 (51%)^f^	12 (1,11–13)^f^	Linguistic abilities (Vocabulary and Similarities from WISC-III short form) Visuospatial abilities (block design and picture arrangement from WISC-III short form)	IQ (all tests), verbal IQ, and performance IQ; multivariate linear regression models	No association between AgeAccelHorvath and IQ/verbal IQ/performance IQ	Age Sex/gender Genetic ancestry (PCs) Cell type composition Birth weight Gestational age Parity Delivery mode Maternal age BMI at delivery Maternal smoking status Alcohol/glycyrrhizin use during pregnancy Education of parents	Low	
Chouliaras *et al.*, 2018 (41)	Horvath 1 Hannum	PBMCs, 450K	CS	Whitehall II Imaging substudy from Whitehall II cohort, United Kingdom, mixed ethnicities (majority White)	47 (17%)	73 (6)	General cognition (MoCA)	General cognition (MoCA sum score); multivariate linear regression models	No association between all AgeAccels and general cognition	Age Sex/gender Cell type composition Smoking Alcohol consumption Premorbid IQ Technical variation (chip and position on array)	Low	23 CN participants and 24 participants with MCI included EWAS to identify differentially methylated probes/regions for cognitive impairment also available
Belsky *et al.*, 2018 (40)	Horvath 1 Hannum Weidner (124)	Blood, 450K	CS and LONGL (∼12 y)	Subsample from Dunedin Multidisciplinary Health and Development Study, New Zealand, mixed ethnicities (majority White)	CS: 818 (NA for specific analysis) LONGL: 743 (NA for specific analysis)	NA for specific analysis	Crystallized intelligence (Information, Similarities, Vocabulary) Fluid intelligence (Digit Symbol Coding, Arithmetic, Block Design, Picture Completion) All measures from WAIS-IV and WISC-R depending on assessment age	Cognition composite score (WAIS-IV at T2 38 y), IQ change since childhood (WAIS-IV at 38 y − average of WISC-R at 7, 9, 11, and 13 y) and separate analysis for IQ subtests; multivariate linear regression models	Higher DNAmAgeWeidner at T2 associated with worse cognition at T2 (nominally with worse cognitive change from childhood) Effects possibly driven by crystallized intelligence Higher Horvath epigenetic ticking (T2 − T1) associated with worse cognition at T2 (nominally for Weidner epigenetic ticking)	Sex/gender BMI	Low	Epigenetic ticking calculated by DNAmAge difference between 2 time points (DNAmAge at 38 y − DNAmAge at 26 y) No major differences when including smoking or SES as covariates in CS analysis
Starnawska *et al.*, 2017 (39)	Horvath 1 Hannum	Blood, 450K	CS and LOGNL (∼10–13 y)	MADT study, Denmark, mixed ethnicities (majority White)	486 [243 monozygotic twin pairs] (46%)	T1: NA (46–67) T2: 70 (6,55–79)	Verbal fluency (Category Fluency Animals) Verbal episodic memory (Immediate and Delayed Word Recall) Working memory (DSF/DSB)	Cognitive composite score representing verbal fluency, immediate word recall, delayed word recall, processing speed, attention, and working memory; paired analysis with multivariate linear regression models, unpaired analysis with multivariate linear mixed-effects regression models	Higher DeltaAgeHorvath at T2 nominally associated with worse cognition at T2 (unpaired approach), otherwise no association between all AgeAccels/DeltaAges and change in cognition over time or cognition at T2 (both approaches)	Age Sex/gender	Moderate	DeltaAge defined as DNAmAge − chronological age Specific tests not mentioned for processing speed and attention Two analytic approaches: paired analysis for within–twin pair differences: correlation of twin differences in cognition (worse performing twin − better performing twin) and twin differences in DNAm/AgeAccel. Unpaired analysis: all individuals analyzed together adjusting for twin-pair relatedness
Degerman *et al.*, 2017 (38)	Horvath 1	Blood, 450K	CS and LONGL (∼15 y)	Betula study, Sweden, mixed ethnicities (majority White)	52 (40%)	T1: 58 (4,55–65) T2: 73 (4,70–80)	Episodic memory (immediate and delayed recall of visually and orally presented sentences, nouns and word lists)	Composite score representing episodic memory; multivariate linear mixed-effects regression models	Higher DeltaAgeHorvath in averages and decliners episodic memory groups at baseline and follow-up (no change in rate of decline over time)	Influence on DNAmAge tested separately: Education Labor force participation Living with someone Smoking habits *APOE*/*COMT* genotype Cell type composition	Low	DeltaAge defined as DNAmAge − chronological age Analysis performed using categories defined by decline in episodic memory over 15–20y (every 5 years): maintainers (high performance maintained), averages (average decline), and decliners (accelerated decline) Based on 1 SD above/below participant-specific and age-specific rate
Wolf *et al.*, 2016 (37)	Horvath 1 Hannum	Blood, 450K	CS	Military veterans (majority with lifetime PTSD), United States, mixed ethnicities (majority White, 71%)	281 (12%)	32 (8,19–58)	Working memory (DSF/DSB, Stroop Color-Word Interference Test, TMT-B)	Latent variable (from CFA) representing working memory; path models	Higher AgeAccelHannum indirectly associated with worse working memory (via fractional anisotropy in genu of corpus callosum)	Age Sex/gender Genetic ancestry (PCs) Cell type composition	Low	AgeAccelHorvath also estimated but not evaluated for association with cognition AgeAccelHannum associated with lifetime PTSD severity (CFA factor)
Marioni *et al.*, 2015 (36)	Horvath 1 Hannum	Blood, 450K	CS and LONGL (6 y)	Subsample from LBC1936, United Kingdom, White	CS: 920 (NA for specific analysis) LONGL: 273 (NA for specific analysis)	NA for specific analysis	Working memory (Letter Number Sequencing, DSB) Nonverbal reasoning (Matrix Reasoning) Visuospatial abilities (Block Design) Processing speed (Digit Symbol Coding, Symbol Search) All measures from WAIS-III	First PC (from PCA) representing fluid intelligence; multivariate linear regression models at baseline, multivariate linear mixed-effects regression models for LONGL analysis	Higher all AgeAccels nominally associated with worse fluid intelligence at baseline No association between all AgeAccels and decline in fluid intelligence over time	Age Sex/gender White blood cell count	Low	AgeAccel at baseline did not predict cognitive decline over time EWAS to identify differentially methylated probes for fluid intelligence (from PCA) also available
Levine *et al.*, 2015 (35)	Horvath 1	Postmortem brain, 450K	LONGL (mean: 4 y, range: 0–16 y)	Subsamples from ROS or Rush MAP, United States, White	700 (64%)	Enrollment: 81 (7,63–102) Death: 88 (7)	Episodic memory Semantic memory Working memory Processing speed—visuospatial abilities [see details regarding specific cognitive tests in Lynch *et al.*, 2023 (76) above]	Global cognition (averaged from all tests); multivariate linear regression models	Higher DNAmAgeHorvath at death associated with worse prior episodic memory (nominally with global cognition) Higher DNAmAgeHorvath at death associated with worse prior episodic memory and global cognition among participants with AD (nominally with working memory)	Age at death Sex/gender Study	Low	Tissue originated from DLPFC 397 CN participants and 303 participants with AD included AgeAccel calculated by regressing out effects of chronological age and sex Higher AgeAccelHorvath associated with more neuropathological measures Association between DNAmAgeHorvath and cognitive function partially mediated by neuropathological measures

AAD indicates the difference between DNAmAge and chronological age; Zhang 1 indicates the best linear unbiased prediction clock; Zhang 2 indicates elastic net clock.

AAD, age acceleration difference; AD, Alzheimer’s disease; ADHD, attention-deficit/hyperactivity disorder; ADNI, Alzheimer’s Disease Neuroimaging Initiative; AgeAccel, age acceleration; AHAB, Adult Health and Behavior; AIBL, Australian Imaging, Biomarkers and Lifestyle; ANCOVA, analysis of covariance; ARIC, Atherosclerosis Risk in Communities; ASPREE, Aspirin in Reducing Events in the elderly; BASE-II, Berlin Aging study II; BBHI, Barcelona Brain Health Initiative; BeCOME, Biological Classification of Mental Disorders; BMI, body mass index; bootEGA, bootstrap exploratory graph analysis; BPD, bipolar disorder; CARDIA, Coronary Artery Risk Development in Young Adults; CERAD, Consortium to Establish a Registry for Alzheimer’s Disease; CFA, confirmatory factor analysis; CHDS, Child Health and Development Study; CN, cognitively normal; CS, cross-sectional; CTAAC, Cape Town Adolescent Antiretroviral Cohort; CUIMC, Columbia University Irving Medical Center; CVLT-II, California Verbal Learning Test-II; DCCS, Dimensional Change Card Sort; DEM, dementia; DISPAR, Disparities Study; DLPFC, dorsolateral prefrontal cortex; DNAm, DNA methylation; DNAmAge, DNA methylation age; DNAmTL, DNA methylation-based estimator of telomere length; DSB, Digit Span Backward; DSF, Digit Span Forward; DSST, Digit Symbol Substitution Test; DunedinPACE, Dunedin pace of aging calculated from the epigenome; DunedinPoAm, Dunedin pace of aging calculated from methylation; EBMT, East Boston Memory Test; ECHO, Epidemiology of Cognitive Health Outcomes; EEAA, extrinsic epigenetic age acceleration; ELSPAC, European Longitudinal Study of Pregnancy and Childhood; EPIC, Illumina MethylationEPIC array; ESCAPE, Effects of Cognitive Aging, Physiology, And Emotion; ETS, Emory Twin Study; FGDS, Female Growth and Development Study; GA:SFHS, Generation Scotland: Scottish Family Health Study; GendAge, Sex- and Gender-Sensitive Prevention of Cardiovascular and Metabolic Disease in Older Adults in Germany; GLAKU, Glycyrrhizin in Licorice; GrimAge2, GrimAge version 2; HANDLS, Healthy Aging in Neighborhoods of Diversity across the Life Span; HC, healthy control; HCAP, Harmonized Cognitive Assessment Protocol; HELIX, Human Early Life Exposome; Horvath 1, Horvath pantissue clock; Horvath 2, SkinBloodClock; HRS, Health and Retirement Study; HVLT-R, Hopkins Verbal Learning Test-Revised; IEAA, intrinsic epigenetic age acceleration; KFACS, Korean Frailty and Aging Cohort Study; LBC1936, Lothian Birth Cohort 1936; LONGL, longitudinal; MADT, Middle-Aged Danish Twin; MAP, Rush Memory and Aging Project; MCI, mild cognitive impairment; MDD, major depressive disorder; MMSE, Mini-Mental State Examination; MoCA, Montreal Cognitive Assessment; NA, not available; NART, National Adult Reading Test; NCDS, National Child Development Study; NEPAL, Neuromodulatory Examination of Pain and Mobility Across the Life span; NEPSY-II, Developmental Neuropsychological Assessment, 2^nd^ ed; NSAID, nonsteroidal anti-inflammatory drug; NSHD, National Survey of Health and Development; oRoB, overall risk of bias; PACC, Preclinical Alzheimer Cognitive Composite; PBMC, peripheral blood mononuclear cell; PCA, principal component analysis; PTSD, posttraumatic stress disorder; RAVLT, Rey’s Auditory Verbal Learning Test; ROS, Religious Orders Study; SATSA, Swedish Adoption/Twin Study of Aging; SCZ, schizophrenia; SES, socioeconomic status; TICS, Telephone Interview for Cognitive Status; TILDA, Irish Longitudinal Study on Aging; TMT, Trail Making Test; VBS, Venous Blood Study; VCAP, Virginia Cognitive Aging Project; VITAL-DEP, vitamin D and OmegA-3 Trial-Depression End point Prevention; VULDE, Underlying Mechanisms of Vulnerability to Depression; WAIS-III, Wechsler Adult Intelligence Scale; WAIS-R, Wechsler Adult Intelligence Scale-revised; WASI, Wechsler Abbreviated Scale of Intelligence; WHIMS, Women’s Health Initiative Memory Study; WHIMS-YA, WHIMS of Younger Women; WISC, Wechsler Intelligence Scale for Children; WISC-R, Wechsler Intelligence Scale for Children-Revised; WMS-III, Wechsler Memory Scale-III; WMS-R, Wechsler Memory Scale-Revised; WTAR, Wechsler Test of Adult Reading.

a Information relates to relevant analysis of associations between epigenetic aging and cognition and may differ from the original publication.

b If information available. In studies with mixed ethnicities, percentage of largest group noted; all values were rounded.

c If information available. All values were rounded.

d Negative findings reported only if positive findings were not present (CS and LONGL analysis treated separately).

e Median reported.

f Information not available for specific analysis but taken from the whole cohort due to minor differences in sample size.

### Assessment of Evidence

A purpose-guided assessment of selected studies, including relevant information for the associations between epigenetic age and cognition, is summarized in Table 1. Whenever available, relevant information regarding study characteristics (cohort characteristics: name, country, and general ethnic composition), tissues and measurement platforms of epigenetic signal (DNA methylation), tools of epigenetic age estimation, investigated cognitive constructs/domains/tests, analysis features (mean, SD, and range of age; sample size; sex/gender; analysis design; statistical methods; outcomes; control variables), and relevant significant findings were incorporated. Additionally, overall risk of bias (RoB) assessment and comments aimed at helping the reader were included.

To allow harmonized descriptive analysis and interpretation, the following approaches were defined: 1) When cohort information was not provided for the subsample used for specific analysis of interest, for studies with minor deviations in sample size (defined as <100 participants for cohorts with >100 participants and <50% of participants for cohorts with <100 participants), information was taken from the whole cohort (and noted in Table 1) and otherwise considered missing. 2) Study population was considered psychiatric if participants with psychiatric disorders were included. 3) Demographic data at baseline were used for longitudinal analysis (if not otherwise specified). 4) Findings for the most complete statistical models were reported, especially due to a strongly differing number of included covariates across studies. 5) A covariate was included in the list of control variables even if an outcome was adjusted for it before inclusion in a multivariate statistical model. 6) Because correction for multiple testing was handled very differently across studies, whenever no explicit correction for multiple testing was mentioned, and results clearly did not overcome a Bonferroni-type correction (based on the number of performed tests), the association was considered nominal. 7) Epigenetic age acceleration (AgeAccel, a residual from regressing estimated epigenetic age on chronological age) is considered a proxy for biological aging (29,30). Therefore, we reported findings related to epigenetic age, delta age, or similar constructs only when AgeAccel was not available. 8) Various terms were used for general cognition in the original publications. We used the term “general cognition” whenever the Mini-Mental State Examination, Montreal Cognitive Assessment, or similar screening tests for general cognitive performance were involved. 9) Cognitive tests were occasionally allocated to different cognitive domains or theoretical cognitive constructs by authors. To enhance comparability, acknowledging that a cognitive test rarely addresses a single cognitive domain, we assigned these tests to common associated cognitive domains and included test names and corresponding cognitive batteries whenever possible (e.g., category fluency measures encompass several domains such as verbal fluency, semantic memory, executive function, and processing speed but were allocated to the executive function domain). Due to incomplete reporting and readability issues, detailed test variables were not included in the table.

### Assessment of RoB

Two reviewers (NY and JF/VNK) applied the Agency for Healthcare Research and Quality checklist (31) (see the Supplement for a complete list) independently to each included study to assess the RoB. This tool was chosen to allow broad applicability to different possible study designs. Possible evaluations were yes, no, or unclear. Overall RoB was defined depending on the number of available “yes” evaluations (total score) and was categorized in the following manner: high (0–4 items), moderate (5–7 items), or low (8–11 items). If an item was not applicable, the score was reduced accordingly. Discrepancies in judgments for any item were resolved by discussion to reach a consensus and if needed were resolved by a third person (EBB, PF).

### Data Synthesis

For data synthesis, we used a narrative synthesis approach. Due to substantial heterogeneity in methodology (covariate use and statistical modeling), reporting, and variables of interest (cognitive tests and epigenetic age assessment), a meta-analysis was not performed. We used descriptive statistics (measures of central tendency/variability or frequency) to describe the data. We evaluated self-reported demographic information (age, percentage of female participants, and ethnic group) across the selected studies. Whenever multiple cohorts were included in a study, the data of the largest cohort were used. To avoid overrepresentation, when sample sizes varied between different tests in a single study, the smallest sample size was considered. Further analyses concerned number of publications per year and study characteristics (study design, inclusion of participants with psychiatric disorders, used tissues, and epigenetic age estimation tools). Mean annual growth rates of published articles were calculated by averaging growth rates (percentage of change) in number of articles per year (although it was not yet over at the time of the literature search, the year 2024 was considered a full year).

To evaluate how often cognition was associated with epigenetic age, we chose a 2-step approach. First, the analysis considered any type of epigenetic age variables (epigenetic age, delta ages, or age differences and epigenetic age acceleration) and any type of cognitive assessment (nominal associations were also included). Next, we performed a more robust analysis, aimed at the investigation of epigenetic age acceleration (as an established proxy for biological aging) (29,30) together with general cognitive constructs and/or specific cognitive domains across selected studies. For the purposes of this analysis, we defined a stricter strategy. Because composite scores or sum scores encompassing several cognitive domains in some studies (often constructed differently) complicated comparability, those studies were excluded. Furthermore, only direct AgeAccel associations were considered (see a detailed list of excluded studies/variables in the Supplement). To further enhance robustness and comparability, nominal associations not surviving correction for multiple testing were considered not associated. Finally, although expressing slightly different concepts, verbal IQ and performance IQ were used as indices of crystallized intelligence and fluid intelligence, respectively.

All statistical analyses were conducted in R version 4.0.4 (32).

## Results

### Article Inclusion and RoB Assessment

**Figure 1:**
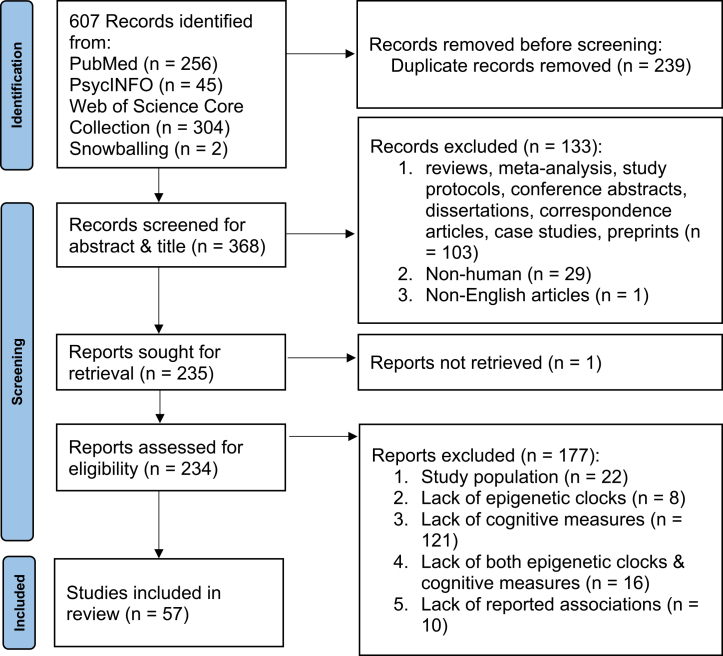
Preferred Reporting Items for Systematic Reviews and Meta-Analyses (PRISMA) flow diagram of search and selection summary.

We identified 57 articles (23, 24, 25,33, 34, 35, 36, 37, 38, 39, 40, 41, 42, 43, 44, 45, 46, 47, 48, 49, 50, 51, 52, 53, 54, 55, 56, 57, 58, 59, 60, 61, 62, 63, 64, 65, 66, 67, 68, 69, 70, 71, 72, 73, 74, 75, 76, 77, 78, 79, 80, 81, 82, 83, 84, 85, 86) matching the eligibility criteria (Figure 1), extracted their data, and summarized them qualitatively (Table 1). During the selection procedure, no conflicts occurred among reviewers, and minor discrepancies were resolved through discussion to reach a consensus. Most studies presented a low RoB (53/57, 93%) (Table S1).

### Rising Interest Over Time

From 2015 (the year of the first included study), there was an overall increase in the number of articles published annually (average growth rate of 30%). More than half of the studies (*n* = 32, 56%) were published during the last 3 evaluated years alone (the total time range was 10 years) (Figure S1).

### General Characteristics

**Figure 2:**
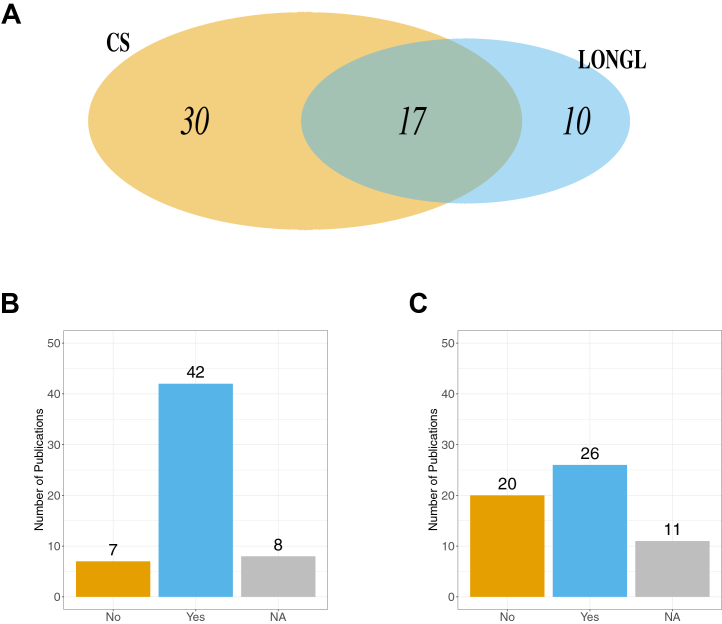
General characteristics and demographics of included studies. **(A)** Number of cross-sectional (CS) analyses (brown) and longitudinal (LONGL) analyses (blue) and studies with both (overlap) are presented. **(B)** Studies with White ethnicity in the majority of participants. **(C)** Studies with female sex/gender in the majority of participants. NA, not available for relevant analysis.

In most selected studies, a cross-sectional analysis was conducted (*n* = 47, 82%), whereas a longitudinal analysis was performed in only 27 studies (47%), with 37,516 and 15,551 participants, respectively (Figure 2A). In 17 studies, both cross-sectional and longitudinal analyses were performed. Most longitudinal analyses (21/27, 78%) were performed during the last 5 evaluated years. Six of the studies (11%) included participants with psychiatric disorders, and most of these (5/6 studies) were published during the last 3 evaluated years.

### Demographics

The average of reported mean (SD) of age was higher in participants included in longitudinal analyses (64 [5] years) as opposed to cross-sectional analyses (54 [5] years). In most of the studies with available information, most participants self-identified as ethnically White (42/49, 86%) (Figure 2B) and female sex/gender (26/46, 57%) (Figure 2C).

### Epigenetic Age Estimators and Tissues

**Figure 3:**
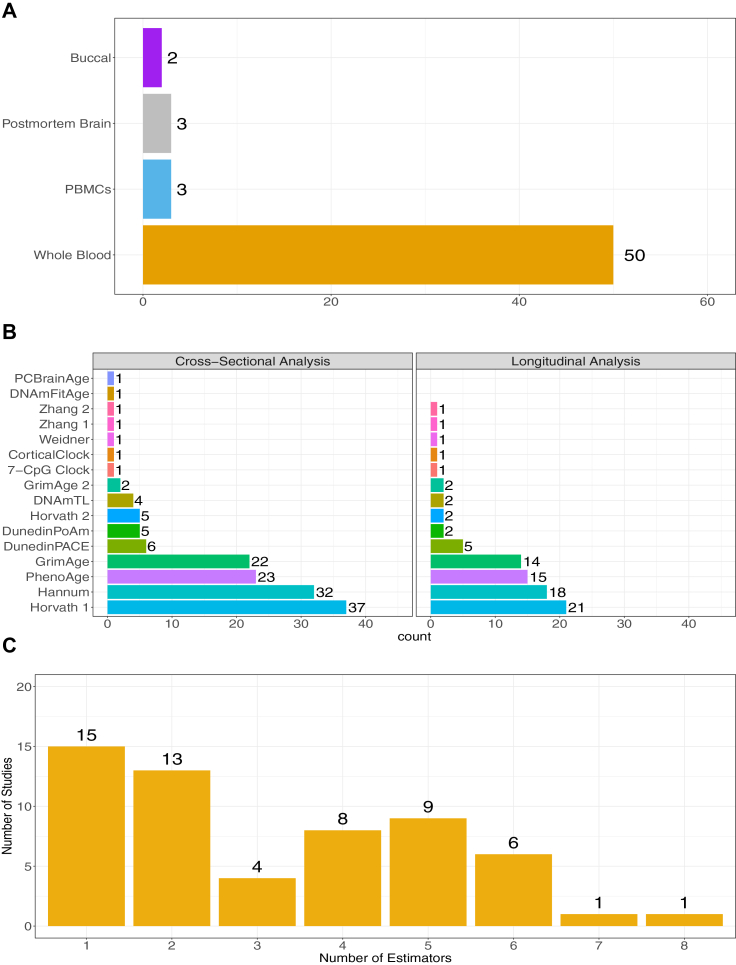
Epigenetic age estimators and investigated tissues of included studies. **(A)** Number of studies investigating a specific tissue. **(B)** Number of epigenetic estimators applied in studies divided by cross-sectional analyses (left) and longitudinal analyses (right). **(C)** Distribution of number of epigenetic age estimators per study across evaluated studies. PBMC, peripheral blood mononuclear cell.

Overall, 16 different tools of epigenetic age estimation (see Table 1 for a complete list) were used in 4 different tissues/cellular compounds, with peripheral whole blood as the most common tissue of interest (peripheral whole blood *n* = 50, peripheral blood mononuclear cells *n* = 3, postmortem brain tissue *n* = 3, buccal tissue *n* = 2) (Figure 3A). Only one study included >1 tissue and compared epigenetic age measured in peripheral blood and in buccal tissue (65). The most common investigated tool of epigenetic age estimation in both analysis designs was the Horvath Pantissue Clock (referred to here as Horvath 1) (87), followed by the Hannum clock (88)—both so called first-generation clocks (trained on chronological age). Next were 2 second-generation clocks (trained on biological aging–related features), PhenoAge (89) and GrimAge (90), followed by 2 third-generation clocks (trained on longitudinal data to obtain a pace of aging estimate), DunedinPACE (86) and DunedinPoAm (91) (Figure 3B). The number of epigenetic age estimators varied across studies. While almost half of the studies (28/57, 49%) included 1 or 2 estimators, others incorporated up to 8 different estimators of epigenetic age (Figure 3C).

### Epigenetic Age and Cognitive Function

The direction of all significant associations related worse cognitive performance to increased epigenetic age measurement, thus showing the expected direction of effects, with the exception of one study (44). Positive associations between any assessment of epigenetic age and any cognitive function were found in 43 studies (75%). Among these studies, 5 included psychiatric patients (including patients with schizophrenia, bipolar disorder, major depressive disorder, and lifetime posttraumatic stress disorder [PTSD]).

**Figure 4:**
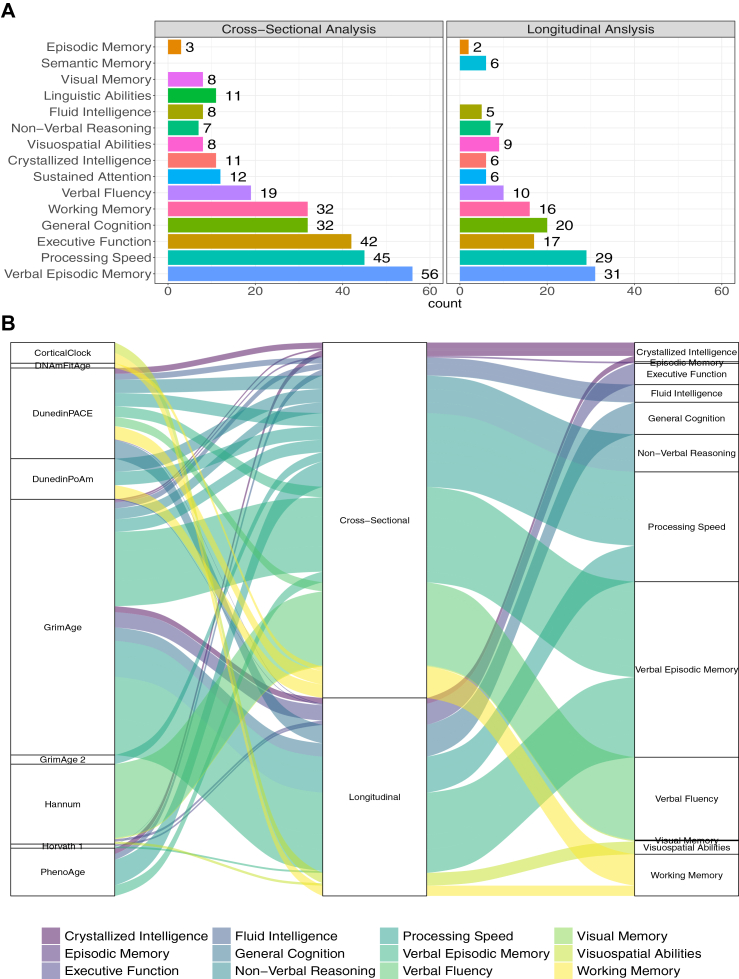
Epigenetic age acceleration and cognitive function. **(A)** Number of cognitive constructs investigated across evaluated studies divided by analysis design (left: cross-sectional; right: longitudinal). **(B)** An overview of significant associations between epigenetic age accelerations and cognitive constructs/domains. The relationship is guided by the middle column to indicate the analysis design of the specific association (cross-sectional vs. longitudinal). Each color represents a cognitive construct/domain to help orientation. The thickness of lines is determined by the total number of participants across studies.

To specifically investigate associations between epigenetic age acceleration and general cognitive constructs and/or specific cognitive domains, data for the analysis were selected as described above (38 studies could be included). In these studies, 15 cognitive constructs were investigated, with verbal episodic memory being the most common construct in both cross-sectional and longitudinal analyses, followed by processing speed and executive function (see Figure 4A for complete results). Twenty-one studies (55%) showed at least 1 significant association (see Table S2 for a complete list). Of these, 2 studies included participants with psychiatric disorders (23,45). Considering all tested associations, most of the reported associations were nonsignificant (yes = 29/9, 9% and no = 265/90, 1% for cross-sectional analyses; yes = 19/11, 6% and no = 145/88, 4% for longitudinal analyses) (Figures S2 and S3, respectively). Significant associations were found for 9 epigenetic age estimators and 12 cognitive constructs (Figure 4B and Table S2). AgeAccelGrim showed significant associations with various cognitive domains in both cross-sectional and longitudinal analyses, presenting the highest number of significant associations across studies (*n* = 19) (Figure 4B). The two most common associations were between AgeAccelGrim and verbal episodic memory (*n* = 7) or processing speed (*n* = 4). DunedinPACE was associated with the highest number of cognitive constructs (9/12, 75%) (Figure 4B) including general cognition, crystallized intelligence, fluid intelligence, executive function, nonverbal reasoning, processing speed, verbal episodic memory, verbal fluency, and working memory. AgeAccelGrim was associated with the second-highest number of cognitive constructs (8/12, 67%) (Figure 4B) including general cognition, crystallized intelligence, fluid intelligence, executive function, nonverbal reasoning, processing speed, verbal episodic memory, and episodic memory. These 2 estimators presented a high ratio of significant associations while being investigated in a considerable number of studies (AgeAccelGrim: 9/25 or 36%; DunedinPACE: 19/72 or 26%). AgeAccels derived from second- and third-generation clocks (except for AgeAccelPheno: 4/82 or 5%) generally presented a higher rate of significant associations as opposed to first-generation clocks (see Table S3 for significant association rates). Finally, executive function was associated with the highest number of epigenetic age estimators (Horvath 1, Hannum, GrimAge, DunedinPoAm, and DunedinPACE).

## Discussion

Identification of individuals at risk for accelerated age-related cognitive decline via peripheral blood biomarkers could allow early detection and intervention, particularly for patients with psychiatric disorders who might be at increased risk (16). Beyond risk detection, such a tool could facilitate targeted neuropsychological assessments, cognitive training, and guided interventions to preserve functioning across the lifespan and in disease. This is especially relevant as several risk factors for cognitive decline, including physical activity, diet, and cognitive training, are modifiable (92) and may have even stronger detrimental effects at younger ages (93).

When we were reviewing the literature, we discovered a growing interest in both general and psychiatric populations. Overall, the vast majority of studies were performed in the general population. Studies including individuals with psychiatric diagnoses were considerably smaller in size (*N*_max_ = 313) as opposed to studies with individuals from the general population (*N*_max_ = 6795). Across all studies, while evidence for an association between measures of cognitive decline and accelerated epigenetic aging was present, no single epigenetic aging construct stood out with convincing consistency (i.e., a high number of available studies and repeated associations across all current studies that would survive corrections for multiple testing). Therefore, larger studies are needed to understand the robustness of the associations and to determine whether specific epigenetic age measures can be utilized as clinical biomarkers.

Our data synthesis suggests that the second- and third-generation clocks AgeAccelGrim and DunedinPACE may be the most promising estimators of epigenetic age acceleration in the context of cognitive aging. While epigenetic age estimators that were developed earlier, such as Horvath 1 and Hannum, were tested frequently (over 100 times), they showed very low rates of significant associations. This is consistent with previous review of epigenetic aging in neurodegenerative diseases and related outcomes, in which first-generation clocks exhibited less consistent associations (94).

AgeAccelGrim was most frequently associated with verbal episodic memory and processing speed. Evidence for these findings is supported by large cumulative sample sizes in both cross-sectional (*n* = 4545 for verbal episodic memory and *n* = 769 for processing speed) and longitudinal (*n* = 4789 for verbal episodic memory and *n* = 2207 for processing speed) analyses. Interestingly, one of the latter studies was performed with trauma-exposed military veterans, most of whom had a lifetime diagnosis of PTSD (23). AgeAccelGrim, and even more so DunedinPACE, seem to capture diverse signals of cognitive function (in both general and wide range constructs of specific cognitive domains). This is promising, as epigenetic age acceleration was associated with cognitive functions typically affected within the framework of cognitive aging. This included general constructs such as fluid intelligence, as well as specific cognitive domains such as verbal episodic memory, reasoning, executive function, and most importantly processing speed. Interestingly, many of the same domains are also affected in various psychiatric disorders (12, 13, 14, 15).

In summary, several significant associations were found between assessments of epigenetic age and cognitive function, most encouragingly for AgeAccelGrim and DunedinPACE. While evidence in the context of psychiatric disorders exists, this area remains understudied. Current findings require replication and validation in the future, because inconsistencies in the current literature limit data synthesis and interpretation. The main reasons include variability in reporting, study design, choice of epigenetic age assessments and cognitive tests, statistical approaches, and covariate adjustment. Because heterogeneity may arise partially from the analysis of studies not specifically designed to address this research question, the field would benefit from prospective, tailored study designs. Although the topics discussed below are unlikely to change substantially with studies published beyond the time scope of this review, this limitation should be considered when interpreting the findings. Next, we highlight sources of heterogeneity and remaining gaps and recommend directions for future research.

Despite recent trends toward longitudinal analysis, most studies relied on cross-sectional analyses design. Notably, longitudinal analyses usually did not assess both cognitive function and epigenetic age at all time points. Epigenetic aging was mostly assessed at baseline (except for studies in postmortem brain tissue), and cognitive function was in some cases only available at a different time point. To establish a link between a biomarker and a future state or change in cognitive function, repeated measures of both variables are essential. This is to avoid distortion by the absence of cognitive assessment at baseline (95) and to allow characterization of within-individual change (e.g., direction and extent). Usually, follow-up periods were long (2–38 years). While this is ideal for developing early biomarkers of decline, studies with shorter intervals and more frequent sampling might help characterize the dynamics of the association, which remain largely unexplored. Shorter assessment periods might also avoid concerns regarding lower qualitative equivalency of some cognitive constructs across young and old age groups (96) and reduce statistical variation introduced by environmental influence on related epigenetic processes. Another important aspect and source of heterogeneity is high within-person variability of performance in cognitive tests (97), which could be addressed by more frequent assessment of cognitive function, for example via including cognitive ecological momentary assessment (98). Among other advantages [see (99) for a systematic review], such approaches may enhance the detection of subtle cognitive changes relevant to daily function (100) and might complement traditional assessments by considering temporal dynamics of external influences (e.g., sleep, social context, mood) on cognitive performance while maintaining high between- and within-person reliability and construct validity (99,101). This may also lead to the discovery of new facets as shown by Zavala *et al.* (33), who found an association between age acceleration and increased interindividual variability in working memory and processing-speed tasks.

Second, studies used a broad range of various epigenetic age estimators to assess a great variety of cognitive constructs. Additionally, the number of epigenetic age estimators included in each study varied greatly, with some studies investigating up to 8 different tools. Moreover, a very large panel of varying cognitive tests was used and operationalized differently (single tests or mixed constructs either by summing multiple scores or building latent factors to represent cognitive domains). The rationale for selecting specific estimators or cognitive tests was mostly unexplained, likely reflecting the exploratory nature of many analyses. Another important challenge in the field of epigenetic aging is an insufficient understanding of underlying biological mechanisms (19,102). Epigenetic clocks detect a variety of biological processes, e.g., DNA damage, DNA repair, cell division, cell type composition, metabolic stress, but they remain incompletely understood [see (30) for a review]. It is very likely that different estimators of epigenetic age capture distinct or partially overlapping facets of the aging process, resulting in heterogeneous associations with age-related decline in function and disease (103). Such exploratory approaches are prone to alpha inflation (multiple testing) and produce a high number of negative associations, which could increase uncertainty. Reducing the number of tests could be achieved by focusing on the most affected cognitive domains in the theoretical framework of cognitive aging or developing clocks specifically trained to capture cognitive aging. Sex-specific associations, which remain poorly studied, may represent an additional source of heterogeneity (84).

Third, heterogeneity may also stem from interindividual differences in cognitive reserve (CR), maintenance, and compensation [see (104) for a review]. These concepts describe the ability of the brain to accumulate, functionally adapt, and maintain cognitive performance and (at least partially) overcome disease-related or age-related damage (105). The biological substrate of CR is not yet fully clear but may arise from flexibility in brain activity and functional connectivity (105). Although some studies have adjusted for CR-related variables (e.g., premorbid IQ or education), the concept itself has not yet been exhaustively explored in the context of epigenetic aging and cognitive decline. For example, a recent study proposed a mediating effect of AgeAccel on the relationship between dynamic brain connectivity and cognition in young adult women (106). Integrating brain imaging and CR measures could be beneficial, especially in psychiatric patients, given a peak age of onset during young adulthood for many psychiatric disorders (107), a time period when CR is considered high.

Fourth, systematic inclusion of important covariates was lacking across studies. Because epigenetics and cognitive performance are known to be influenced by genetics, environmental factors, socioeconomic status, and lifestyle (108, 109, 110), consistent adjustment is essential. However, there are currently no standard adjustment strategies. While this may depend on the research questions, proposed covariates include sex, smoking, alcohol use, physical activity, genetic information to account for genetic heterogeneity, socioeconomic status, comorbidities, and cell type composition (111).

Finally, general considerations related to biomarker properties must be acknowledged (19,112). In particular, epigenetic signal could be strongly influenced by variation in cell type proportions (30,113), but only approximately half of the studies adjusted for this covariate. Test-retest reliability is another important issue (19). This could be improved by newer approaches such as principal-component clocks (114) or third-generation epigenetic estimators such as DunedinPACE (86).

Beyond these considerations, broader future directions could integrate biological age estimators from various molecular compounds beyond epigenetics (so called omics such as metabolomics, transcriptomics, and proteomics) or magnetic resonance imaging–based brain aging. This could potentially enhance predictive performance (30,115). Lastly, a better understanding of normal brain health and cognitive aging will advance insights into pathological aging processes.
