# Supplementary material for: Epigenetic Aging and Cognitive Performance in General and Psychiatric Populations: A Systematic Review and Narrative Synthesis
Source: Biol Psychiatry Glob Open Sci. 2026 Mar 30;6(4):100726. doi: 10.1016/j.bpsgos.2026.100726 (PMC13273795; doi:10.1016/j.bpsgos.2026.100726)
Supplement: Supplemental Text, Figures S1–S3, and Tables S1–S3 [file mmc1.pdf]

SUPPLEMENTARY INFORMATION

Epigenetic Aging and Cognitive Performance in General and Psychiatric Populations: A Systematic Review and Narrative Synthesis

Yusupov *et al.*

Content:

1. Figure S1
2. Figure S2
3. Figure S3
4. Table S1
5. Table S2
6. Table S3
7. Deviations from preregistered protocol
8. Agency for Healthcare Research and Quality (ARHQ) checklist
9. Studies/Study variables not included in analysis due to use of composite cognitive scores, cognitive sum scores, joint cognitive domains, lack of AgeAccel associations, use of indirect associations

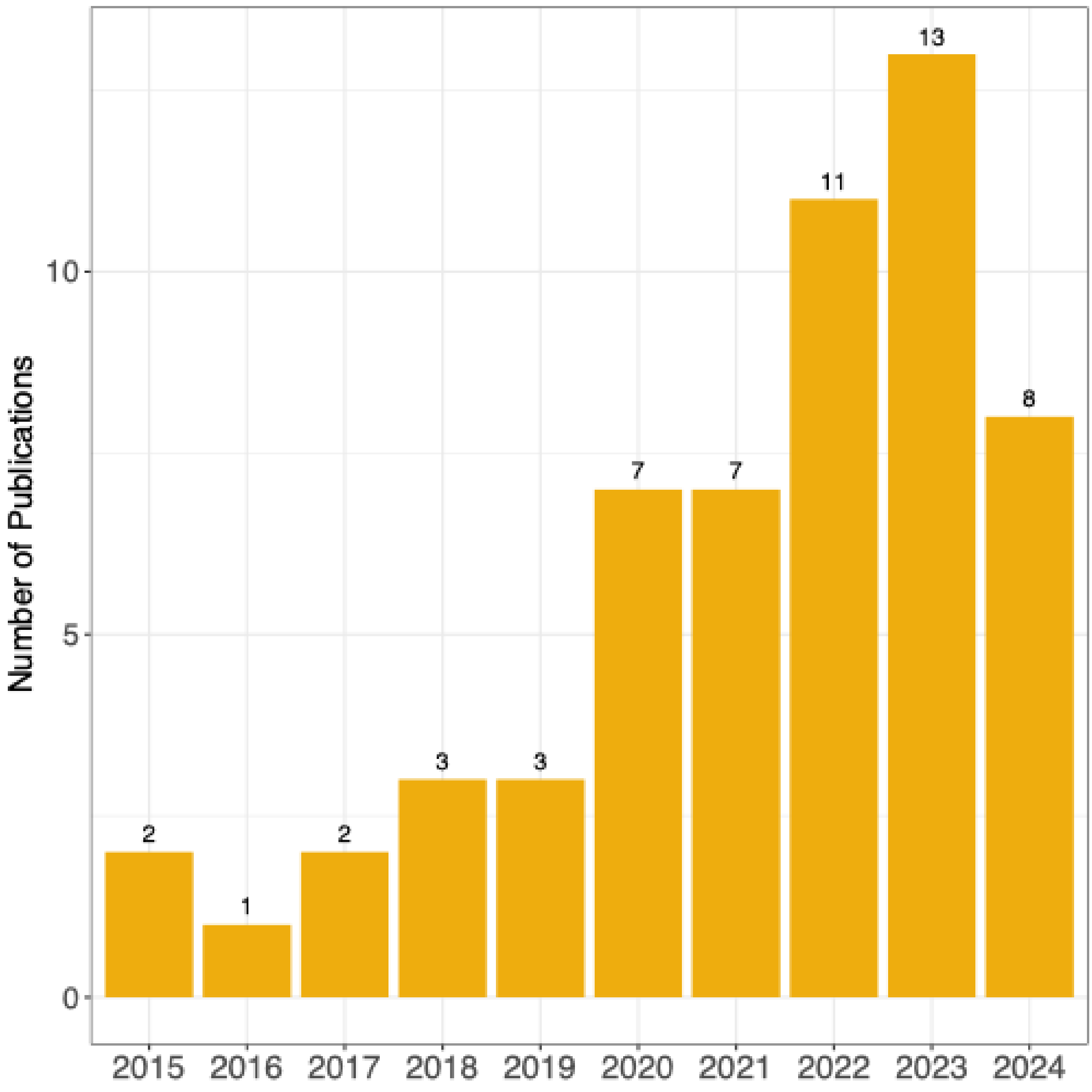

Figure S1: Distribution of articles by year of publication.
Number of published studies per year among the articles included in the review.

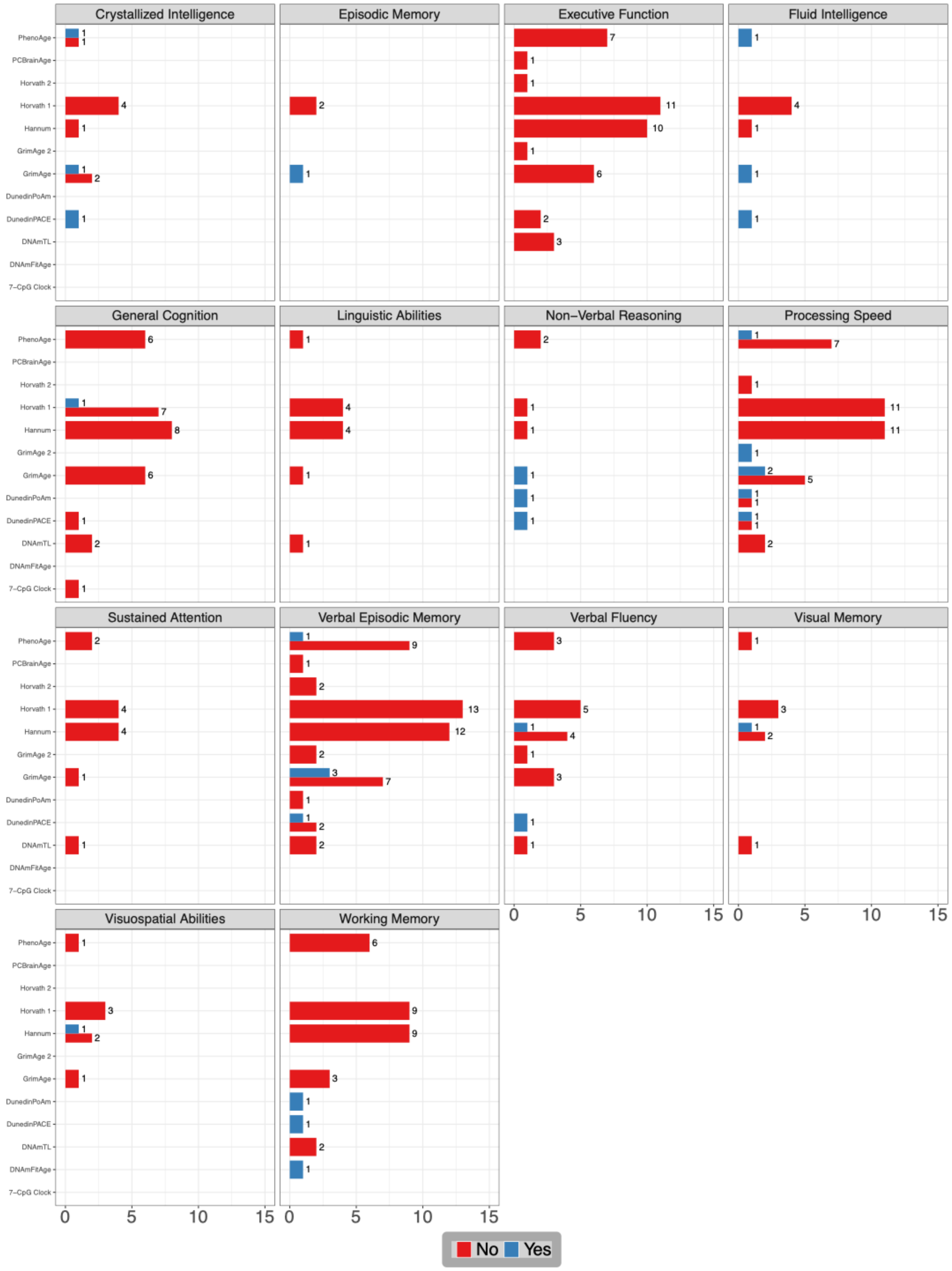

Figure S2: Cross-sectional associations between epigenetic age acceleration and cognitive function.

Displayed is the number of associations between epigenetic age acceleration and cognitive function for each epigenetic age estimator separated by cognitive constructs/domains (blue: significant association; red: non-significant association).

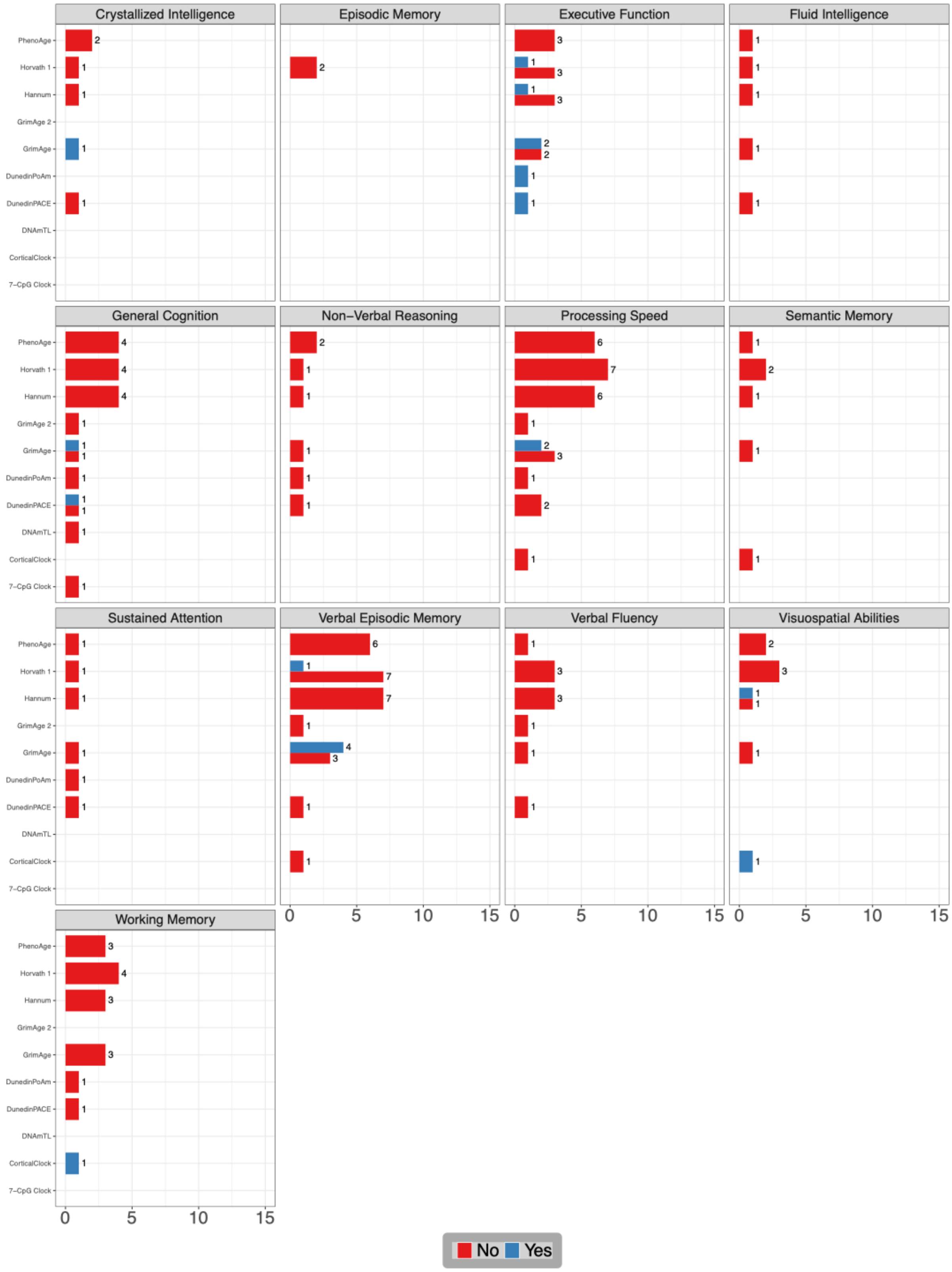

Figure S3: Longitudinal associations between epigenetic age acceleration and cognitive function.

Displayed is the number of associations between epigenetic age acceleration and cognitive function for each epigenetic age estimator separated by cognitive constructs/domains (blue: significant association; red: non-significant association).

Nr.	Study	1	2	3	4	5	6	7	8	9	10	11	Overall RoB
1	Zavala et al., 2024	Yes	Yes	Yes	Yes	NA	Yes	Yes	Yes	Yes	Yes	NA	Low
2	Wolf et al., 2024	Yes	Yes	Yes	Yes	No	Yes	Yes	Yes	Yes	Yes	Yes	Low
3	Ware et al., 2024	Yes	Yes	Yes	Yes	No	No	Yes	Yes	Yes	Yes	NA	Low
4	Phyo et al., 2024	Yes	Yes	Yes	Yes	No	Yes	Yes	Yes	Yes	Yes	Yes	Low
5	Nguyen et al., 2024	Yes	Yes	Yes	Yes	No	Yes	Yes	Yes	Yes	Yes	Yes	Low
6	Graves et al., 2024	Yes	Yes	No	No	No	Yes	Yes	Yes	Yes	Yes	NA	Low
7	Engvig et al., 2024	Yes	Yes	Yes	Yes	No	Yes	Yes	Yes	Yes	Yes	NA	Low
8	Chen et al., 2024	Yes	Yes	Yes	Yes	No	Yes	Yes	Yes	Yes	Yes	NA	Low
9	Vyas et al., 2023	Yes	Yes	Yes	Yes	No	Yes	Yes	Yes	Yes	Yes	Yes	Low
10	Stephan et al., 2023	Yes	Yes	Yes	Yes	No	Yes	Yes	Yes	Yes	Yes	Yes	Low
11	Robinson et al., 2023	Yes	Yes	Yes	Yes	No	Yes	Yes	Yes	Yes	Yes	NA	Low
12	O'Shea et al., 2023	Yes	Yes	Yes	Yes	No	Yes	Yes	Yes	Yes	Yes	NA	Low
13	Mareckova et al., 2023	Yes	Yes	Yes	Yes	No	Yes	Yes	Yes	Yes	Yes	NA	Low

14	Lynch MT et al., 2023	Yes	Yes	Yes	Yes	Yes	Yes	Yes	Yes	Yes	No	NA	Low
15	Lynch M et al., 2023	Yes	Yes	Yes	Yes	No	Yes	Yes	Yes	Yes	Yes	NA	Low
16	Li et al., 2023	Yes	Yes	No	No	No	Yes	NA	Yes	NA	Yes	Yes	Low
17	Jokai et al., 2023	Yes	No	No	No	No	Yes	NA	Yes	Yes	Yes	NA	Moderate
18	Heany et al., 2023	Yes	Yes	Yes	Yes	No	Yes	Yes	Yes	Yes	Yes	Yes	Low
19	Felt et al., 2023	Yes	Yes	Yes	Yes	No	Yes	Yes	Yes	Yes	Yes	NA	Low
20	Faul et al., 2023	Yes	Yes	Yes	Yes	No	Yes	Yes	Yes	Yes	Yes	Yes	Low
21	Arpawong et al., 2023	Yes	Yes	Yes	Yes	No	Yes	Yes	Yes	Yes	Yes	NA	Low
22	Zheng et al., 2022	Yes	Yes	Yes	Yes	No	Yes	Yes	Yes	Yes	Yes	NA	Low
23	Vetter et al., 2022	Yes	Yes	Yes	Yes	No	Yes	Yes	Yes	Yes	Yes	Yes	Low
24	Sugden et al., 2022	Yes	Yes	Yes	Yes	No	Yes	Yes	Yes	Yes	Yes	Yes	Low
25	Sommerer et al., 2022	Yes	Yes	Yes	Yes	No	Yes	Yes	Yes	Yes	Yes	Yes	Low
26	Segura et al., 2022	Yes	Yes	Yes	Yes	No	Yes	Yes	Yes	Yes	Yes	NA	Low
27	Reed et al., 2022	Yes	Yes	Yes	Yes	No	Yes	Yes	Yes	Yes	Yes	Yes	Low

28	Pérez et al., 2022	Yes	Yes	Yes	Yes	No	Yes	Yes	Yes	Yes	Yes	Yes	Low
29	Milicic et al., 2022	Yes	Yes	Yes	Yes	No	Yes	Yes	Yes	Yes	Yes	Yes	Low
30	Lima et al., 2022	Yes	Yes	No	Yes	No	Yes	Yes	Yes	Yes	Yes	NA	Low
31	Krivonosov et al. 2022	Yes	Yes	No	No	No	Yes	Yes	No	No	No	NA	Moderate
32	Belsky et al. 2022	Yes	Yes	Yes	Yes	No	Yes	Yes	Yes	Yes	Yes	Yes	Low
33	Vaccarino et al., 2021	Yes	Yes	Yes	Yes	No	Yes	Yes	Yes	Yes	Yes	Yes	Low
34	Shiau et al., 2021 (1)	Yes	Yes	Yes	Yes	No	Yes	Yes	Yes	No	Yes	Yes	Low
35	Shiau et al., 2021 (2)	Yes	Yes	Yes	Yes	No	Yes	Yes	Yes	No	Yes	Yes	Low
36	Park et al., 2021	Yes	Yes	Yes	Yes	No	Yes	Yes	Yes	NA	Yes	NA	Low
37	McCrory et al., 2021	Yes	Yes	Yes	Yes	No	Yes	NA	Yes	Yes	Yes	NA	Low
38	Hillary et al., 2021	Yes	Yes	Yes	Yes	No	Yes	NA	Yes	Yes	Yes	Yes	Low
39	Grodstein et al., 2021	Yes	Yes	Yes	Yes	No	Yes	Yes	Yes	Yes	Yes	Yes	Low
40	Wiesman et al., 2020	Yes	Yes	Yes	Yes	No	Yes	Yes	Yes	Yes	Yes	NA	Low
41	Maddock et al., 2020	Yes	Yes	Yes	Yes	No	Yes	Yes	Yes	Yes	Yes	Yes	Low

42	Li et al., 2020	Yes	Yes	Yes	Yes	No	Yes	Yes	Yes	Yes	Yes	Yes	Low
43	Hoare et al., 2020	Yes	Yes	Yes	Yes	No	Yes	Yes	Yes	Yes	Yes	NA	Low
44	Hillary et al., 2020	Yes	Yes	Yes	Yes	No	Yes	Yes	Yes	Yes	Yes	NA	Low
45	Bressler et al., 2020	Yes	Yes	Yes	Yes	No	Yes	Yes	Yes	Yes	Yes	Yes	Low
46	Beydoun et al., 2020	Yes	Yes	Yes	Yes	No	Yes	Yes	Yes	Yes	Yes	Yes	Low
47	Vyas et al., 2019	Yes	Yes	Yes	Yes	No	Yes	No	No	No	Yes	NA	Moderate
48	Stevenson et al., 2019	Yes	Yes	Yes	Yes	No	Yes	No	Yes	No	Yes	Yes	Low
49	Cruz-Almeida et al., 2019	Yes	Yes	Yes	Yes	No	Yes	Yes	Yes	Yes	Yes	NA	Low
50	Suarez et al., 2018	Yes	Yes	Yes	Yes	No	Yes	Yes	Yes	No	Yes	NA	Low
51	Chouliaras et al., 2018	Yes	Yes	Yes	Yes	No	Yes	Yes	Yes	No	Yes	NA	Low
52	Belsky et al., 2018	Yes	Yes	Yes	Yes	No	Yes	Yes	Yes	Yes	Yes	Yes	Low
53	Starnawska et al., 2017	Yes	Yes	Yes	Yes	No	Yes	NA	Yes	No	No	No	Moderate
54	Degerman et al., 2017	Yes	Yes	Yes	Yes	No	Yes	Yes	Yes	No	No	Yes	Low
55	Wolf et al., 2016	Yes	Yes	Yes	Yes	No	Yes	Yes	Yes	Yes	Yes	NA	Low

56	Marioni et al., 2015	Yes	Yes	Yes	Yes	No	Yes	No	Yes	No	Yes	Yes	Low
57	Levine et al., 2015	Yes	Yes	Yes	Yes	No	Yes	Yes	Yes	Yes	Yes	NA	Low

Table S1: Quality assessment.
Overall risk of bias was categorized to "high" (0-4 items), "moderate" (5-7 items) or "low" (8-11 items). Abbreviations: NA, not applicable; RoB, risk of bias.

**Study & year**	**Population**	**Tissue**	**N**	**Estimator**	**Design**	**cognitive domain**
**wolf_2024**	Psychiatric	Blood	159	GrimAge	Longl	Verbal episodic_memory
**phyo_2024**	General	Blood	560	GrimAge 2	Cs	Processing speed
**phyo_2024**	General	Blood	560	DunedinPACE	Cs	Verbal fluency
**nguyen_2024**	General	Blood	1134	DunedinPACE	Longl	General cognition
**graves_2024**	General	Blood	103	GrimAge	Cs	Processing speed
**graves_2024**	General	Blood	103	GrimAge	Cs	Episodic memory
**graves_2024**	General	Blood	103	GrimAge	Cs	Crystallized intelligence
**chen_2024**	General	Blood	359	PhenoAge	Cs	Crystallized intelligence
**chen_2024**	General	Blood	359	PhenoAge	Cs	Fluid intelligence
**chen_2024**	General	Blood	359	GrimAge	Cs	Fluid intelligence
**chen_2024**	General	Blood	359	DunedinPACE	Cs	Crystallized intelligence
**chen_2024**	General	Blood	359	DunedinPACE	Cs	Fluid intelligence
**chen_2024**	General	Blood	359	GrimAge	Longl	Crystallized intelligence
**stephan_2023**	General	Blood	2423	GrimAge	Longl	Verbal episodic memory
**oshea_2023**	General	Blood	1171	GrimAge	Cs	Verbal episodic memory
**lynch_m_2023**	General	Blood	1814	GrimAge	Cs	Verbal episodic memory
**jokai_2023**	General	Blood	294	DNAmFitAge	Cs	Working memory
**zheng_2022**	General	Blood	890	GrimAge	Longl	Executive function
**zheng_2022**	General	Blood	890	GrimAge	Longl	Verbal episodic memory
**zheng_2022**	General	Blood	890	GrimAge	Longl	Processing speed
**sugden_2022**	General	Blood	649	PhenoAge	Cs	Verbal episodic memory
**sugden_2022**	General	Blood	649	DunedinPACE	Cs	Verbal episodic memory
**reed_2022**	General	Blood	48	GrimAge	Longl	Executive function
**reed_2022**	General	Blood	48	DunedinPoAm	Longl	Executive function
**reed_2022**	General	Blood	48	DunedinPACE	Longl	Executive function
**belsky_2022**	General	Blood	814	DunedinPoAm	Cs	Non verbal reasoning
**belsky_2022**	General	Blood	814	DunedinPoAm	Cs	Working memory
**belsky_2022**	General	Blood	814	DunedinPoAm	Cs	Processing speed
**belsky_2022**	General	Blood	814	DunedinPACE	Cs	Non verbal reasoning

**belsky_2022**	General	Blood	814	DunedinPACE	Cs	Working memory
**belsky_2022**	General	Blood	814	DunedinPACE	Cs	Processing speed
**belsky_2022**	General	Blood	814	GrimAge	Longl	General cognition
**vaccarino_2021**	General	PBMCs	114	Horvath 1	Longl	Executive function
**vaccarino_2021**	General	PBMCs	114	Horvath 1	Longl	Verbal episodic memory
**hoare_2020**	General	Blood	44	Hannum	Cs	Visual memory
**hoare_2020**	General	Blood	44	Hannum	Cs	Visuospatial abilities
**bressler_2020**	General	Blood	4510	Hannum	Cs	Verbal fluency
**beydoun_2020**	General	Blood	147	Hannum	Longl	Visuospatial abilities
**beydoun_2020**	General	Blood	147	Hannum	Longl	Executive function
**vyas_2019**	Psychiatric	Blood	23	Horvath 1	Cs	General cognition
**maddock_2020**	General	Blood	1560	PhenoAge	Cs	Processing speed
**maddock_2020**	General	Blood	1560	GrimAge	Cs	Verbal episodic memory
**maddock_2020**	General	Blood	1317	GrimAge	Longl	Verbal episodic memory
**maddock_2020**	General	Blood	1317	GrimAge	Longl	Processing speed
**hillary_2021**	General	Blood	666	GrimAge	Cs	Processing speed
**hillary_2021**	General	Blood	666	GrimAge	Cs	Non verbal reasoning
**grodstein_2021**	General	PB	633	Cortical clock	Longl	Working memory
**grodstein_2021**	General	PB	633	Cortical clock	Longl	Visuospatial abilities

Table S2: List of significant associations between epigenetic age acceleration and cognitive function.
Presented are for each significant association the study, year of publication, investigated population and tissue, sample size, estimator and cognitive construct/domain as well as analysis design. Abbreviations: Pb, postmortem brain; Longl, longitudinal; Cs, cross-sectional.

**Estimator**	**No**	**Yes**	**Total**	**Significant associations rate (%)**
**7-CpG Clock**	2	0	2	0
**CorticalClock**	3	2	5	40
**DNAmFitAge**	0	1	1	100
**DNAmTL**	16	0	16	0
**DunedinPACE**	16	9	25	36
**DunedinPoAm**	7	4	11	36
**GrimAge**	53	19	72	26
**GrimAge 2**	8	1	9	11
**Hannum**	101	5	106	5
**Horvath 1**	120	3	123	2
**Horvath 2**	4	0	4	0
**PCBrainAge**	2	0	2	0
**PhenoAge**	78	4	82	5

Table S3: Rates of significant associations.
Presented are for each epigenetic age acceleration the number of significant, non-significant and total investigated associations with any cognitive construct as well as the rates of significant associations (in percent).

7. Deviations from preregistered protocol

Following deviations were conducted while performing this review:

1. Vera B. Karlbauer joined the review team to assist Julia Fietz in her part of the full text review and data extraction step as an independent second reviewer. Additionally, VBK contributed to critical evaluation and discussion of the results.
2. Interventional studies were not excluded initially, but later due to the defined eligibility criteria. Non were included in the final review.
3. As original peer-reviewed studies were to be included, dissertations and correspondence articles were not included in the review, although not stated explicitly initially.
4. In light of a great heterogeneity among included studies (e.g., cognitive assessments, outcome measurements, covariates inclusion and statistical analysis), no clear recommendations could have been made according to the recommendations suggested by the Grading of Recommendations Assessment, Development and Evaluation (GRADE) (Guyatt et al., 2008).

8. ARHQ checklist:
   1. Define source of information (survey, record, review)
   2. List inclusion and exclusion criteria for exposed and unexposed subjects* (cases and controls) or refer to previous publications
   3. Indicate time period used for identifying patients
   4. Indicate whether or not subjects were consecutive if not population-based
   5. Indicate if evaluators of subjective components of study were masked to other aspects of the status of the participants
   6. Describe any assessments undertaken for quality assurance purposes (e.g., test/retest of primary outcome measurements)
   7. Explain any patient exclusions from analysis
   8. Describe how confounding was assessed and/or controlled
   9. If applicable, explain how missing data were handled in the analysis
   10. Summarize patient response rates and completeness of data collection
   11. Clarify what follow-up, if any, was expected and the percentage of patients for which incomplete data or follow-up was obtained

   *Comment: general inclusion/exclusion criteria for subjects assessed whenever case/control status was not defined

9. Studies/Study variables not included in analysis due to:

   A. Composite scores: Findings related to composite score from Phyo et al., Heany et al., Hoara et al. and Grodstein et al. (only non-composite results included) (Grodstein et al., 2021; Heany et al., 2023; Hoare et al., 2020; Phyo et al., 2024),

Li et al. (Li et al., 2020), Faul et al. (Faul et al., 2023), Hillary et al. (Hillary et al., 2020), Starnawska et al. (Starnawska et al., 2017), findings related to IQ from Mareckova et al. and Suarez et al. (Mareckova et al., 2023; Suarez et al., 2018), Marioni et al. (Marioni et al., 2015), Milicic et al. (Milicic et al., 2022), Felt et al. (Felt et al., 2023), findings related to composite scores for longitudinal analysis from Hillary et al. (Hillary et al., 2021).

B. Sum scores: Findings related to composite sum score from Vyas et al. and Lynch M et al. (only rest of results included) (M. Lynch et al., 2023; Vyas et al., 2023), Ware at al. (Ware et al., 2024), Lynch MT et al. (M. T. Lynch et al., 2023).

C. joint domains scores: Reasoning/Visuospatial abilities laten variable from Graves et al. (Graves et al., 2024).

D. Lack of AgeAccel associations: Zavala et al. (Zavala et al., 2024), findings from longitudinal analysis from Vyas et al. (Vyas et al., 2023), Robinson et al. (Robinson et al., 2023), Cruz-Almeida et al. (Cruz-Almeida et al., 2019), Belsky et al. (Belsky et al., 2018), Degerman et al. (Degerman et al., 2017), findings for Horvath clock from Heany et al. (Heany et al., 2023), Arpawong et al. (Arpawong et al., 2023), Levine et al. (Levine et al., 2015), Krivonosov et al. (Krivonosov et al., 2022), Wiesman et al. (Wiesman et al., 2020).

E. Use of indirect associations: Wolf et al. (Wolf et al., 2016).

References

Arpawong, T. E., Klopack, E. T., Kim, J. K., & Crimmins, E. M. (2023). ADHD genetic burden associates with older epigenetic age: mediating roles of education, behavioral and sociodemographic factors among older adults. *CLINICAL EPIGENETICS*, *15*(1), Article 67. https://doi.org/10.1186/s13148-023-01484-y

Belsky, D. W., Moffitt, T. E., Cohen, A. A., Corcoran, D. L., Levine, M. E., Prinz, J. A., Schaefer, J., Sugden, K., Williams, B., Poulton, R., & Caspi, A. (2018). Eleven Telomere, Epigenetic Clock, and Biomarker-Composite Quantifications of Biological Aging: Do They Measure the Same Thing? *AMERICAN JOURNAL OF EPIDEMIOLOGY*, *187*(6), 1220–1230. https://doi.org/10.1093/aje/kwx346

Cruz-Almeida, Y., Sinha, P., Rant, A., Huo, Z. G., Fillingim, R. B., & Foster, T. (2019). Epigenetic aging is associated with clinical and experimental pain in community-dwelling older adults. *MOLECULAR PAIN*, *15*, Article 1744806919871819. https://doi.org/10.1177/1744806919871819

Degerman, S., Josefsson, M., Adolfsson, A. N., Wennstedt, S., Landfors, M., Haider, Z., Pudas, S., Hultdin, M., Nyberg, L., & Adolfsson, R. (2017). Maintained memory in aging is associated with young epigenetic age. *Neurobiology of Aging*, *55*, 167–171. https://doi.org/10.1016/j.neurobiolaging.2017.02.009

Faul, J. D., Kim, J. K., Levine, M. E., Thyagarajan, B., Weir, D. R., & Crimmins, E. M. (2023). Epigenetic-based age acceleration in a representative sample of older Americans: Associations with aging-related morbidity and mortality. *PROCEEDINGS OF THE NATIONAL ACADEMY OF SCIENCES OF THE UNITED STATES OF AMERICA*, *120*(9), Article e2215840120. https://doi.org/10.1073/pnas.2215840120

Felt, J. M., Yusupov, N., Harrington, K. D., Fietz, J., Zhang, Z. Y., Sliwinski, M. J., Ram, N., Become Working, G., Meaney, M. J., Putnam, F. W., Noll, J. G., Binder, E. B., Shenk, C. E., & O'Dornell, K. J. (2023). Epigenetic age acceleration as a biomarker for impaired cognitive abilities in adulthood following early life adversity and psychiatric disorders. *Neurobiology of Stress*, *27*, Article 100577. https://doi.org/10.1016/j.ynstr.2023.100577

Graves, A. J., Danoff, J. S., Kim, M., Brindley, S. R., Skyberg, A. M., Giamberardino, S. N., Lynch, M. E., Straka, B. C., Lillard, T. S., Gregory, S. G., Connelly, J. J., & Morris, J. P. (2024). Accelerated epigenetic age is associated with whole-brain functional connectivity and impaired cognitive performance in older adults. *SCIENTIFIC REPORTS*, *14*(1), Article 9646. https://doi.org/10.1038/s41598-024-60311-3

Grodstein, F., Lemos, B., Yu, L., Klein, H. U., Iatrou, A., Buchman, A. S., Shireby, G. L., Mill, J., Schneider, J. A., De Jager, P. L., & Bennett, D. A. (2021). The association of epigenetic clocks in brain tissue with brain pathologies and common aging phenotypes. *NEUROBIOLOGY OF DISEASE*, *157*, Article 105428. https://doi.org/10.1016/j.nbd.2021.105428

Guyatt, G. H., Oxman, A. D., Vist, G. E., Kunz, R., Falck-Ytter, Y., Alonso-Coello, P., Schunemann, H. J., & Group, G. W. (2008). GRADE: an emerging consensus on rating quality of evidence and strength of recommendations. *BMJ*, *336*(7650), 924–926. https://doi.org/10.1136/bmj.39489.470347.AD

Heany, S. J., Levine, A. J., Lesosky, M., Phillips, N., Fouche, J. P., Myer, L., Zar, H. J., Stein, D. J., Horvath, S., & Hoare, J. (2023). Persistent accelerated epigenetic ageing in a longitudinal cohort of vertically infected HIV-positive adolescents. *Journal of Neurovirology*, *29*(3), 272–282. https://doi.org/10.1007/s13365-023-01130-6

Hillary, R. F., Stevenson, A. J., Cox, S. R., McCartney, D. L., Harris, S. E., Seeboth, A., Higham, J., Sproul, D., Taylor, A. M., Redmond, P., Corley, J., Pattie, A., Hernández, M. D. V., Muñoz-Maniega, S., Bastin, M. E., Wardlaw, J. M., Horvath, S., Ritchie, C. W., Spires-Jones, T. L.,…Marioni, R. E. (2021). An epigenetic predictor of death captures multi-modal measures of brain health. *Molecular Psychiatry*, *26*(8), 3806–3816. https://doi.org/10.1038/s41380-019-0616-9

Hillary, R. F., Stevenson, A. J., McCartney, D. L., Campbell, A., Walker, R. M., Howard, D. M., Ritchie, C. W., Horvath, S., Hayward, C., McIntosh, A. M., Porteous, D. J., Deary, I. J., Evans, K. L., & Marioni, R. E. (2020). Epigenetic measures of ageing predict the prevalence and incidence of leading causes of death and disease burden. *Clin Epigenetics*, *12*(1), 115. https://doi.org/10.1186/s13148-020-00905-6

Hoare, J., Stein, D. J., Heany, S. J., Fouche, J. P., Phillips, N., Er, S., Myer, L., Zar, H. J., Horvath, S., & Levine, A. J. (2020). Accelerated epigenetic aging in adolescents from low-income households is associated with altered development of brain structures. *METABOLIC BRAIN DISEASE*, *35*(8), 1287–1298. https://doi.org/10.1007/s11011-020-00589-0

Krivonosov, M. I., Kondakova, E. V., Bulanov, N. A., Polevaya, S. A., Franceschi, C., Ivanchenko, M. V., & Vedunova, M. V. (2022). A new cognitive clock matching phenotypic and epigenetic ages. *TRANSLATIONAL PSYCHIATRY*, *12*(1), Article 364. https://doi.org/10.1038/s41398-022-02123-5

Levine, M. E., Lu, A. T., Bennett, D. A., & Horvath, S. (2015). Epigenetic age of the pre-frontal cortex is associated with neuritic plaques, amyloid load, and Alzheimer's

disease related cognitive functioning. *AGING-US*, *7*(12), 1198–1211. https://doi.org/10.18632/aging.100864

Li, X., Ploner, A., Wang, Y., Magnusson, P. K., Reynolds, C., Finkel, D., Pedersen, N. L., Jylhävä, J., & Hägg, S. (2020). Longitudinal trajectories, correlations and mortality associations of nine biological ages across 20-years follow-up. *ELIFE*, *9*. https://doi.org/10.7554/eLife.51507

Lynch, M., Arpawong, T. E., & Beam, C. R. (2023). Associations Between Longitudinal Loneliness, DNA Methylation Age Acceleration, and Cognitive Functioning. *JOURNALS OF GERONTOLOGY SERIES B-PSYCHOLOGICAL SCIENCES AND SOCIAL SCIENCES*, *78*(12), 2045–2059. https://doi.org/10.1093/geronb/gbad128

Lynch, M. T., Taub, M. A., Farfel, J. M., Yang, J. Y., Abadir, P., De Jager, P. L., Grodstein, F., Bennett, D. A., & Mathias, R. A. (2023). Evaluating genomic signatures of aging in brain tissue as it relates to Alzheimer's disease. *SCIENTIFIC REPORTS*, *13*(1), Article 14747. https://doi.org/10.1038/s41598-023-41400-1

Mareckova, K., Pacinkova, A., Marecek, R., Sebejova, L., Izakovicova Holla, L., Klanova, J., Brazdil, M., & Nikolova, Y. S. (2023). Longitudinal study of epigenetic aging and its relationship with brain aging and cognitive skills in young adulthood. *Frontiers in Aging Neuroscience*, *15*. https://doi.org/10.3389/fnagi.2023.1215957

Marioni, R. E., Shah, S., McRae, A. F., Ritchie, S. J., Muniz-Terrera, G., Harris, S. E., Gibson, J., Redmond, P., Cox, S. R., Pattie, A., Corley, J., Taylor, A., Murphy, L., Starr, J. M., Horvath, S., Visscher, P. M., Wray, N. R., & Deary, I. J. (2015). The epigenetic clock is correlated with physical and cognitive fitness in the Lothian Birth Cohort 1936. *INTERNATIONAL JOURNAL OF EPIDEMIOLOGY*, *44*(4), 1388–1396. https://doi.org/10.1093/ije/dyu277

Milicic, L., Vacher, M., Porter, T., Doré, V., Burnham, S. C., Bourgeat, P., Shishegar, R., Doecke, J., Armstrong, N. J., Tankard, R., Maruff, P., Masters, C. L., Rowe, C. C., Villemagne, V. L., Laws, S. M., Alzheimer's Dis Neuroimaging, I., & Australian Imaging Biomarkers, L. (2022). Comprehensive analysis of epigenetic clocks reveals associations between disproportionate biological ageing and hippocampal volume. *GEROSCIENCE*, *44*(3), 1807–1823. https://doi.org/10.1007/s11357-022-00558-8

Phyo, A. Z. Z., Wu, Z. M., Espinoza, S. E., Murray, A. M., Fransquet, P. D., Wrigglesworth, J., Woods, R. L., & Ryan, J. (2024). Epigenetic age acceleration and cognitive performance over time in older adults. *ALZHEIMER'S & DEMENTIA: DIAGNOSIS, ASSESSMENT & DISEASE MONITORING*, *16*(3), Article e70010. https://doi.org/10.1002/dad2.70010

Robinson, O., Lau, C. E., Joo, S., Andrusaityte, S., Borras, E., de Prado-Bert, P., Chatzi, L., Keun, H. C., Grazuleviciene, R., Gutzkow, K. B., Maitre, L., Martens, D. S., Sabido, E., Siroux, V., Urquiza, J., Vafeiadi, M., Wright, J., Nawrot, T. S., Bustamante, M., & Vrijheid, M. (2023). Associations of four biological age markers with child development: A multi-omic analysis in the European HELIX cohort. *ELIFE*, *12*, Article e85104. https://doi.org/10.7554/eLife.85104

Starnawska, A., Tan, Q., Lenart, A., McGue, M., Mors, O., Børglum, A. D., Christensen, K., Nyegaard, M., & Christiansen, L. (2017). Blood DNA methylation age is not associated with cognitive functioning in middle-aged monozygotic twins. *Neurobiology of Aging*, *50*, 60–63. https://doi.org/10.1016/j.neurobiolaging.2016.10.025

Suarez, A., Lahti, J., Czamara, D., Lahti-Pulkkinen, M., Girchenko, P., Andersson, S., Strandberg, T. E., Reynolds, R. M., Kajantie, E., Binder, E. B., & Raikkonen, K. (2018). The epigenetic clock and pubertal, neuroendocrine, psychiatric, and cognitive outcomes in adolescents. *CLINICAL EPIGENETICS*, *10*, Article 96. https://doi.org/10.1186/s13148-018-0528-6

Vyas, C. M., Sadreyev, R. I., Gatchel, J. R., Kang, J. H., Reynolds, C. F., III, Mischoulon, D., Chang, G., Hazra, A., Manson, J. E., Blacker, D., De Vivo, I., & Okereke, O. I. (2023). Pilot study of second-generation DNA methylation epigenetic markers in relation to cognitive and neuropsychiatric symptoms in older adults. *Journal of Alzheimer's Disease*, *93*(4), 1563–1575. https://doi.org/10.3233/JAD-230093

Ware, E. B., Tejera, C. H., Wang, H. R., Harris, S., Fisher, J. D., & Bakulski, K. M. (2024). Interplay of education and DNA methylation age on cognitive impairment: insights from the Health and Retirement Study. *GEROSCIENCE*. https://doi.org/10.1007/s11357-024-01356-0

Wiesman, A. I., Rezich, M. T., O'Neill, J., Morsey, B., Wang, T. N., Ideker, T., Swindells, S., Fox, H. S., & Wilson, T. W. (2020). Epigenetic Markers of Aging Predict the Neural Oscillations Serving Selective Attention. *Cerebral Cortex*, *30*(3), 1234–1243. https://doi.org/10.1093/cercor/bhz162

Wolf, E. J., Logue, M. W., Hayes, J. P., Sadeh, N., Schichman, S. A., Stone, A., Salat, D. H., Milberg, W., McGlinchey, R., & Miller, M. W. (2016). Accelerated DNA methylation age: Associations with PTSD and neural integrity. *Psychoneuroendocrinology*, *63*, 155–162. https://doi.org/10.1016/j.psyneuen.2015.09.020

Zavala, D. V., Dzikowski, N., Gopalan, S., Harrington, K. D., Pasquini, G., Mogle, J., Reid, K., Sliwinski, M., Graham-Engeland, J. E., Engeland, C. G., Bernard, K., Veeramah, K., & Scott, S. B. (2024). Epigenetic Age Acceleration and Chronological Age: Associations With Cognitive Performance in Daily Life. *JOURNALS OF GERONTOLOGY SERIES A-BIOLOGICAL SCIENCES AND MEDICAL SCIENCES*, *79*(1). https://doi.org/10.1093/gerona/glad242
